# Non-host plant odors influence the tritrophic interaction between tomato, its foliar herbivore *Tuta absoluta* and mirid predator *Nesidiocoris tenuis*

**DOI:** 10.3389/fpls.2023.1014865

**Published:** 2023-03-23

**Authors:** Bashiru Adams, Abdullahi Ahmed Yusuf, Baldwyn Torto, Fathiya Mbarak Khamis

**Affiliations:** ^1^Department of Behavioural and Chemical Ecology, International Centre of Insect Physiology and Ecology (icipe), Nairobi, Kenya; ^2^Department of Zoology and Entomology, University of Pretoria, Hatfield, South Africa

**Keywords:** invasive pest, Asteraceae, terpenes, attractant, repellent, predator

## Abstract

The tomato leafminer, *Tuta absoluta* is a destructive invasive pest of cultivated tomato and other Solanaceae plants, with yield losses of 80-100%. Mirid predators are key natural enemies of *T. absoluta*, but they also feed on host plants in the absence of their prey. Management of *T. absoluta* is a challenge due to its high biotic potential, resistance to many insecticides and the absence of sufficiently adapted auxiliary fauna in its new dispersion zones. Olfaction plays an important role in the tritrophic interaction between tomato, its herbivore pest *T. absoluta* and its mirid predators, which can be influenced by non-host plant odors. However, how non-host odours shape this interaction is poorly understood. Previously, we had demonstrated belowground crop protection properties of certain Asteraceae plants against the root-knot nematode *Meloidogyne incognita*, pest of tomato and other Solanaceae plants. Additionally, Asteraceae plants impact negatively on feeding behavior of above-ground pests of Solanaceae plants, including the greenhouse whitefly (*Trialeurodes vaporariorum*) and green peach aphid (*Myzus persicae*). Here, we tested the hypothesis that foliar volatiles from some of these non-host Asteraceae plants can influence the tomato-*T. absoluta*-mirid predator tritrophic interaction. In olfactometer assays, *T. absoluta* females were attracted to volatiles of the Solanaceae host plants tomato and giant nightshade but avoided volatiles of the Asteraceae plants, blackjack and marigold, and the positive control, wild tomato, when tested alone or in combination with the host plants. Coupled gas chromatography-mass spectrometry analysis showed that host and non-host plants varied in their emission of volatiles, mainly monoterpenes and sesquiterpenes. Random forest analysis combined with behavioral assays identified monoterpenes as the host plant attractive blend to *T. absoluta* and its mirid predator, with sesquiterpenes identified as the non-host plant repellent blend against *T. absoluta*. Contrastingly, the mirid predator was indifferent to the non-host plant repellent sesquiterpenes. Our findings indicate that terpenes influence the tomato-*T. absoluta*-mirid predator tritrophic interaction. Further, our results emphasize the importance of studying crop protection from a holistic approach to identify companion crops that serve multi-functional roles.

## 1 Introduction

Phytophagous insects use olfactory cues to locate their host plants, for feeding, mating, and oviposition (Bawin et al., 2016; Antwi-Agyakwa et al., 2019; Piersanti et al., 2020). The quality and quantity of the phytochemical signal may determine the effectiveness of the interaction with the insect (Bai et al., 2020; Msisi et al., 2020; Antwi-Agyakwa et al., 2021), especially in a host and non-host plant intercrop system. The volatiles released by each plant in the intercropping system can affect the host location by the insect pest in various ways including repellency or eliciting a masking effect on host attractive volatiles. For instance, guava (*Psidium guajava*) and garlic (*Allium sativum*) volatiles decreased attraction of females of the African citrus triozid (*Trioza erytreae*) to its common host plant, rough lemon (*Citrus jambhiri*), attributed to the relative abundance of the sesquiterpenes (*E*)-caryophyllene and (*E*)-4,8-dimethyl-1,3,7-nonatriene (DMNT), benzaldehydes, nonanal and decanal, respectively (Antwi-Agyakwa et al., 2021). Likewise, in a laboratory study, basil (*Ocimum basilicum*) and marigold (*Tagetes patula*) volatiles decreased attraction and reduced fecundity of the green peach aphid (*Myzus persicae*), in the presence of the host plant *Capsicum annuum* attractive volatiles (Dardouri et al., 2021). However, the volatiles responsible for avoidance behavior in the green peach aphid were not identified. Additionally, the monoterpenes linalool and (*Z*)-β-ocimene released from marigold (*T*. *minuta*) and basil were found to repel the greenhouse whitefly (*Trialeurodes vaporariorum*) in the presence of the host plant tomato (*Solanum lycopersicum*) attractive volatiles (Matu et al., 2021). While these examples demonstrate the potential of using non-host plants to reduce pest populations, currently, we lack detailed examples of the use of non-host plants to manage other key insect pests of tomato such as the tomato leafminer *T. absoluta* (Lepidoptera: Gelechiidae).

The tomato leafminer, *T. absoluta* (Lepidoptera: Gelechiidae) is an invasive pest native to South America that was introduced into Spain in 2006 (Desneux et al., 2010; Tonnang et al., 2015). Currently, *T. absoluta* is present in the Middle East, Africa, and Asia (Giorgini et al., 2019). Its main host in the tropics and subtropics is cultivated tomato (Rakha et al., 2017), although it attacks other solanaceous plants including eggplant (*Solanum melongena*), black nightshade (*Solanum nigrum*), pepper (*Capsicum annuum*), potato (*Solanum tuberosum*), and tobacco (*Nicotiana tabacum*) and non-solanaceous plants such as common bean (*Phaseolus vulgaris*) (Bitew, 2018). Thus, secondary hosts make biocontrol difficult. Adult females of *T. absoluta* oviposit on leaves (73%), shoots and flowers (26%) as well as on fruits (1%) of tomatoes (Desneux et al., 2010; Cocco et al., 2013; Illakwahhi and Srivastava, 2017), resulting in yield losses of 80-100% caused by larval feeding in unprotected fields (Illakwahhi and Srivastava, 2017; Giorgini et al., 2019). This compromises the source of livelihood of many farmers in Africa.

Current management techniques of *T. absoluta* include cultural methods, pheromones, biocontrol agents, resistant varieties, and synthetic insecticides (Guedes and Picanço, 2012; Biondi et al., 2018; Giorgini et al., 2019). However, these management methods are not effective because of the cryptic nature of the feeding larvae (Guedes and Picanço, 2012; Ali and Husin, 2017). Use of synthetic chemical insecticides has been the quickest and most efficient method to control the pest, but this is not sustainable due to insecticide resistances of *T. absoluta*, negative effects of insecticides on the environment, non-target organisms and the health of farmers (Sridhar et al., 2019). As such, it has prompted research into safer integrated control approaches that strongly feature the use of biorationals (Giorgini et al., 2019; Tarusikirwa et al., 2020; Erasmus and Van Den Berg, 2021).

Most research on *T. absoluta* has focused on its biological control with predators (Sanchez et al., 2014; Biondi et al., 2018; Sanchez et al., 2018). Among the key predators of *T. absoluta* are the zoophytophagous mirids such as *Macrolophus pygmaeus* (Hemiptera: Miridae), native to Europe and *N. tenuis* (Hemiptera: Miridae), native to the Mediterranean coast (Sanchez et al., 2006; Sanchez et al., 2009; van Lenteren, 2012; Zappalà et al., 2013; Lins et al., 2014; Sanchez et al., 2018; Ayelo et al., 2021). For example, in a greenhouse tomato production study in Europe, *M.pygmaeus* was found to significantly reduced the population of whiteflies *Bemisia tabaci* and *T. absoluta* galleries (Sanchez et al., 2018; Konan et al., 2021). These predators prey on eggs and lower instars of other small arthropods such as aphids, spider mites, and thrips (van Lenteren, 2012; Zappalà et al., 2013; Sylla et al., 2016; Biondi et al., 2018). In the absence of sufficient prey, they feed on leaf mesophylls, ground tissues of stems, inflorescences, and fruits of host plants resulting in significant yield reduction (Moerkens et al., 2016; Sanchez et al., 2018; Konan et al., 2021). Previous studies have demonstrated that the availability of diverse food sources for generalist natural enemies in heterogeneous habitats reduced damage to host plants and attracted more predators compared to monocultures (Tang et al., 2013; Grez et al., 2014). This suggests that altering the habitat of mirid predators by increasing plant diversity can influence their behaviour.

Previous crop protection studies demonstrated that plants in the Asteraceae family contained phytochemicals that suppressed belowground pest parasitism (Torto et al., 2018; Fabrick et al., 2020; Mwamba et al., 2021). For instance, recent studies showed that the root exudates of blackjack (*Bidens pilosa*), marigold (*Tagetes minuta*), pyrethrum (*Chrysanthemum cinerariifolium*), and sweet wormwood (*Artemisia annua*) suppressed host finding in the root-knot nematode (*Meloidogyne incognita*), a pest of tomato and other Solanaceae plants (Conboy et al., 2019; Mwamba et al., 2021; Kihika-Opanda et al., 2022). The monoterpene 1,8-cineole and the sesquiterpene (*E*)-β-farnesene from the root volatiles of blackjack, marigold and sweet wormwood repelled *M. incognita* to reduce infection on the host plant tomato (Mwamba et al., 2021). In another study, ascorbic acid (vitamin C) and 2-hydroxybenzoic acid (aromatic acid) detected in the root exudates of blackjack had a negative impact on egg hatchability and mortality of juveniles by disrupting the chemoreception of *M. incognita* (Kihika-Opanda et al., 2022). As to aboveground interactions, for example, intercropping marigold (*T. minuta*) with tomato reduced the abundance and growth of the greenhouse whitefly (*T*. *vaporariorum*), attributed to the presence of limonene detected in the headspace volatiles of the plant (Conboy et al., 2019). Likewise, the related species *Tagetes erecta* was also found to significantly reduce the population of *Psylliodes chrysocephala* and *Plutella xylostella*, pests of the crop Pak Choi (*Brassica rapa chinensis*) in an intercrop with Pak Choi plant, while attracting more unidentified natural enemies of the pests than Pak Choi in monoculture (Iamba and Homband, 2020).

Here, we have extended our studies to test the hypothesis that foliar volatiles of some of these selected Asteraceae plants including blackjack (*Bidens pilosa*) and marigold (*T. minuta*) (Kihika et al., 2020; Mwamba et al., 2021) can influence the host finding behavior of the above-ground pest, the invasive tomato leafminer *Tuta absoluta*, and its associated mirid predator *N. tenuis* in two Solanaceae host plants, tomato, and giant nightshade. To achieve this, we tested the responses of *T. absoluta* females to volatiles of host plants, and when the host plants were combined with non-host plants, and we compared the chemistry of the odor profiles of these plants using random forest classification followed by behavioral assays and identified the attractive and repellent volatiles. Additionally, we investigated the response of adults of the mirid predator *N. tenuis* of *T. absoluta* to the identified host and non-host plant attractive and repellent volatiles.

## 2 Materials and methods

### 2.1 Plants

Seeds of cultivated tomato, *Solanum lycopersicum* (moneymaker cultivar), and giant nightshade, *Solanum scabrum* were obtained from Simlaw Seeds Company Ltd., Nairobi, Kenya. The seeds were sterilized by dipping them in 1% sodium hypochlorite solution for 3 min to remove seed-borne pathogens that may induce chemical defenses in the plant, and rinsed with distilled water thrice. The sterilized seeds were then sown in nursery trays containing sterilized loamy soil (autoclaved for 30 min at 121°C) in a screenhouse maintained at (25 ± 2°C, 60-70% RH and L12:D12 light/dark photoperiod) at the International Centre of Insect Physiology and Ecology (*icipe*), Duduville Campus, Nairobi, Kenya (1580 m, S 01°13.243’ E 036°53.732’). After germination, the seedlings (2- weeks- old) were transplanted into plastic pots (5 L with 30 cm depth) containing autoclaved loamy soil. The seedlings were watered daily. Wild tomato which was recently found to disrupt the host finding behavior of *T. absoluta* (Miano et al., 2022), was also tested. Wild tomato, *Lycopersicon esculentum* var. *cerasiforme* seedlings were obtained from stock maintained in a screenhouse at *icipe* which was established from seeds obtained from a farmer’s field at Kiangai (S 0° 28’ 59” E 37° 19’ 59”) in Kirinyaga County of Kenya. Wild tomato seedlings were grown using similar procedures as outlined for the two host plants. Cultivated tomato, nightshade, and wild tomato seedlings that were 5-6 weeks old were used in all the experiments. Marigold and blackjack seedlings were also obtained from stocks maintained at *icipe* in a screen house. The seedlings of marigold and blackjack were grown in plastic pots using the same procedures outlined for the other plants. As stated earlier, marigold and blackjack seedlings (5-6 weeks old) were used for the study because repellent compounds that impacted negatively on root-knot nematode in previous studies were released by the plants at these developmental stages (Kihika et al., 2020; Mwamba et al., 2021). The plants used for the experiment were all in the vegetative stages.

### 2.2 Insects

*Tuta absoluta* adults were obtained from a colony maintained (28 ± 2°C, 48% RH and 12:12 L:D light/dark photoperiod) on potted susceptible Cal J tomato variety plants (seeds sourced from Simlaw Seeds Company Ltd., Nairobi, Kenya) in well-ventilated Perspex cages (40 cm × 50 cm × 60 cm) at the insectary section of the Animal Rearing and Quarantine Unit (ARQU) of *icipe*. The colony was established from *T. absoluta* infested tomato plants collected from a farmer’s field in Mwea (S 0° 36′ 31.3″ E 037° 22′ 29.7″) and Naivasha (N 0° 43’ 0.01” E 36° 26’ 9.28”) in December 2020. The moths were fed on 80% honey solution ad libitum. To reduce inbreeding, the colony was rejuvenated every three months by infusing it with freshly infested tomato plants collected from farmer’s fields at Mwea and Naivasha. Gravid females (2-3 days old) were used for all the behavioral assays. To ensure that only gravid females were used in the behavioral assays, only mating mature adult females found in the rearing cage were collected and used for the assays after 24 hr.

The mirid predator was collected from farmers’ field in Mwea (S 0° 36′ 31.3″ E 037° 22′ 29.7″) and were reared on potted Moneymaker tomato variety plants (6-8 weeks old) in Plexiglass cages (40 cm × 50 cm × 60 cm) maintained in a laboratory (25 ± 2°C, 60 ± 5% RH, L12:D12) at the ARQU of *icipe*. The insects were fed on a mixture of non-viable eggs of *Ephestia kuehniella* Zeuler and *Artemia* sp. (Koppert biological system, Veilingweg, Netherlands) twice a week. A total of 10 g of the nonviable eggs were provided in each of the cages per week. Feeding was also supplemented with tomato plants containing viable eggs of *T. absoluta* after 3-4 days exposure of tomato plants to females of the moth. Additionally, the mirid predator was fed on 80% honey solution. Each week the adults that emerged were transferred to another Plexiglass cage to minimize cannibalism on the young nymphs. Adult males and females of the mirid predator (1:1 ratio) between 1-7 days old (F_2_ generation) were used for all the behavioral assays.

### 2.3 Molecular characterization of the mirid predator: DNA extraction and amplification

Whole insects were brought to the *icipe* Arthropod Pathology Unit for processing. Genomic DNA was extracted from each sample using the Isolate II Genomic DNA Kit (Bioline, London, United Kingdom), following the manufacturer’s instructions. The resultant DNA was eluted in a final 50 μl volume then quality and quantity checks were done using the Nanodrop 2000/2000c Spectrophotometer (Thermo Fischer Scientific, Wilmington, USA). Polymerase chain reaction (PCR) was done to amplify the 28S rDNA large ribosomal domain 2 (D2) gene region using LepD2F (5’-AGTCGTGTTGCTTGATAGTGCAG-3’) and LepD2R (5’-TTGGTCCGTGTTTCAAGACGGG-3’) primers (Campbell et al., 1994; Goolsby et al., 2006). The PCR was conducted in a total reaction volume of 20 µL containing 5X My Taq Reaction Buffer (5 mM dNTPs, 15 mM MgCl2, stabilizers and enhancers), 0.5 pmol/µl of each primer, 0.5 mM MgCl2, 0.0625 U/µl My Taq DNA polymerase (Bioline) and 15 ng/µl of DNA template. These reactions were set up in the Nexus Mastercycler gradient (Eppendorf, Germany). The following cycling conditions were used: initial denaturation for 2 min at 95°C, followed by 40 cycles of 30 sec at 95°C, 30 sec annealing at 58.8°C and 1 min at 72°C, then a final elongation step of 10 min at 72°C.

The amplified PCR products were resolved through a 1.2% agarose gel. DNA bands on the gel were analyzed and documented using KETA GL imaging system trans-illuminator (Wealtec Corp, Meadowvale Way Sparks, Nevada, USA). Successfully amplified products were excised and purified using Isolate II PCR and Gel Kit (Bioline) following the manufacturer’s instructions. The purified samples were shipped to Macrogen Europe BV (Meibergreef, Amsterdam, the Netherlands), for paired end sequencing.

### 2.4 28S rDNA sequence analyses

For a conclusive confirmation of the identity of the predator, whole genome sequencing (WGS) of the insect using the DNBseq sequencing platform at BGI Genomics (BGI, Tai Po, N.T, Hong Kong) was done. The mitochondrial COI gene was extracted after a *De-novo* assembly using SPAdes v.3.13.0 (Bankevich et al., 2012) and contigs were identified by BLAST+ v.2.13.0. (Camacho et al., 2009). The COI gene sequence obtained from this study was deposited in GenBank database.

### 2.5 Behavioural responses of *T. absoluta* to host and non-host plant headspace volatiles

The responses of *T. absoluta* to host and non-host plant volatiles were carried out in a Y-tube olfactometer (arm = 10 cm, stem = 14 cm, diameter = 3 cm). Charcoal-purified and humidified air was passed into the two arms of the olfactometer, each at a flow rate of 350 mL/min. An electric-powered air free vacuum pump (Model: DAA-V174-EB, GAST manufacturing company, Benton Harbor, Michigan, United States) was used to suck air out of the Y-tube at a flow rate of 700 mL/min. Because *T. absoluta* is nocturnal, a night condition was simulated by placing a red fluorescent bulb about 2 m above the olfactometer which emitted about 1000 Lux illuminations of red light. To ensure no positional bias in the olfactometer bioassay, each of the two arms was connected to an empty sterilized (12 hr at 100°C) roasting bag (50 cm × 60 cm) (Lifetime Brands Europe Ltd, Valepits Road, Birmingham), and assayed against each other with 60 mated females. The response of *T. absoluta* females to volatiles from host and non-host plants were tested in assays as shown in **Supplementary Table 1**. Host and non-host plant headspace volatiles were offered into the two arms of the Y-tube olfactometer from the roasting bags containing the plants. To ensure that only headspace plant volatiles were released into the roasting bags, the whole pot containing the soil and the plant were covered with aluminum foil to the base of the plant. In each bioassay, 60 gravid females were tested individually, and each insect was given 10 min to make a choice. An insect was considered to have made a choice when it walked at least 5 cm into one of the arms and stayed for two or more minutes. No gravid female was re-used in any of the assay. Six different plants of each species were used for each assay. After every ten females tested, the plants were replaced with new ones. The Y- tube was cleaned after every 5 insects tested and the arms were then switched. All the bioassays were conducted under controlled laboratory conditions (27°C and 70% RH).

### 2.6 Collection of headspace volatiles

Headspace volatiles were collected from a total of five intact host and non-host plants and an empty roasting bag as a control. Prior to collection of volatiles, the plants were allowed to acclimatize to the laboratory conditions for 12 hr. The aerial part of each test plant was covered with a sterilized roasting bag (12 hr at 100°C) (35 cm × 30 cm) (Lifetime Brands Europe Ltd, Valepits Road, Birmingham) and gently held tightly around the base of the stem with a rubber band to create airtight conditions while avoiding injury to the plant. Charcoal-purified air was passed/bulled (400 mL/min) through a glass-tube containing distilled water to humidify the air. Volatiles were pulled (350 mL/min) from the bags through a vacuum tube connected to a Super-Q trap (30 mg) (Analytical Research System, Gainesville, FL) for 24 hr to capture both day and night volatiles. Super-Q-trapped volatiles were eluted with 150 μL gas chromatography (GC) grade dichloromethane (Analytical grade, Sigma-Aldrich, St, Louis, MO) under a stream of nitrogen gas into 1.5 mL glass vials. All the eluted samples were stored at −80°C until used.

### 2.7 Analysis of volatiles

Volatiles were analyzed by gas chromatography-mass spectrometry (GC-MS) on an HP 7890A series gas chromatograph (Agilent Technologies, Wilmington, USA) coupled with an HP 5975C mass spectrometer (Agilent Technologies, Wilmington, USA). The column used was 30 m × 0.25 mm i.d., 0.25 μm Agilent HP-5 MS capillary column. An aliquot (1 µL) of each sample was injected in the splitless mode at an oven temperature of 35°C for 5 min, which gradually increased to 280°C at 10°C/min and held for 10.5 min. The carrier gas was helium at a flow rate of 1.0 ml/min and the mass spectra were acquired at 70 eV within a mass range of 38–550 Daltons (Da) during a scan time of 0.73 scans sec^-1^. Volatiles were identified by comparing their mass spectral data with the GC/MS library data using, (Adams, 1995; NIST, 2008). Authentic standards were used to confirm the identities of compounds where available and they were based on comparison of their mass spectral data and retention times (RTs). Quantification of the identified volatiles was done using calibration curves (peak area vs. concentration) generated by serial dilutions of the authentic standards α-pinene and (*E*)-caryophyllene analyzed under the same GC/MS conditions at five different concentrations (1−500 ng/μL) since it equally yields results as close to the natural situation as possible. The retention indices (RIs) of the compounds were determined using n-alkane standards (C_8_-C_31_) and calculated using the equation described below by van den Dool and Dec. Kratz (1963), and compared with published literature values.


$$\text{RIx}=100{\text{n}}_{0}+100({\text{R}}_{\text{T}}\text{x}-{\text{R}}_{\text{T}}{\text{n}}_{1})/({\text{R}}_{\text{T}}{\text{n}}_{1}-{\text{R}}_{\text{T}}{\text{n}}_{0})$$

Where:

x = the name of the target compound; n_0_ = n-alkane C_n0_H_2n0+2_ directly eluting after x; n_1_ = n-alkane C_n1_H_2n1+2_ directly eluting before x; R_T_ = retention time; R_I_ = retention index.

### 2.8 Chemicals

The synthetic standards including hexanal, α-thujene, α-pinene, camphene, *o*-cymene, sabinene, β-pinene, myrcene, α-terpinene, β-terpinene, terpinolene, decanal, tridecane, pentadecane, cedrol, hexadecane and the solvent dichloromethane were purchased from Sigma Aldrich (USA) whilst δ-2-carene, α-phellandrene, δ-3-carene, *p*-cymene, limonene, β- phellandrene, β-ocimene [mixture of (*E*) and (*Z*)], dihydrotagetone, δ-elemene, β-elemene, α-cedrene, (*E*)-caryophyllene, α-humulene, (*E*, *E*)-α-farnesene were purchased from Merck (France) and were used to confirm the identified compounds. The chemical purity of the synthetic standards except for sabinene (75%) and α-phellandrene (85%) ranged between 90–99%.

### 2.9 Bioassays with synthetic standards

**Figure 1:**
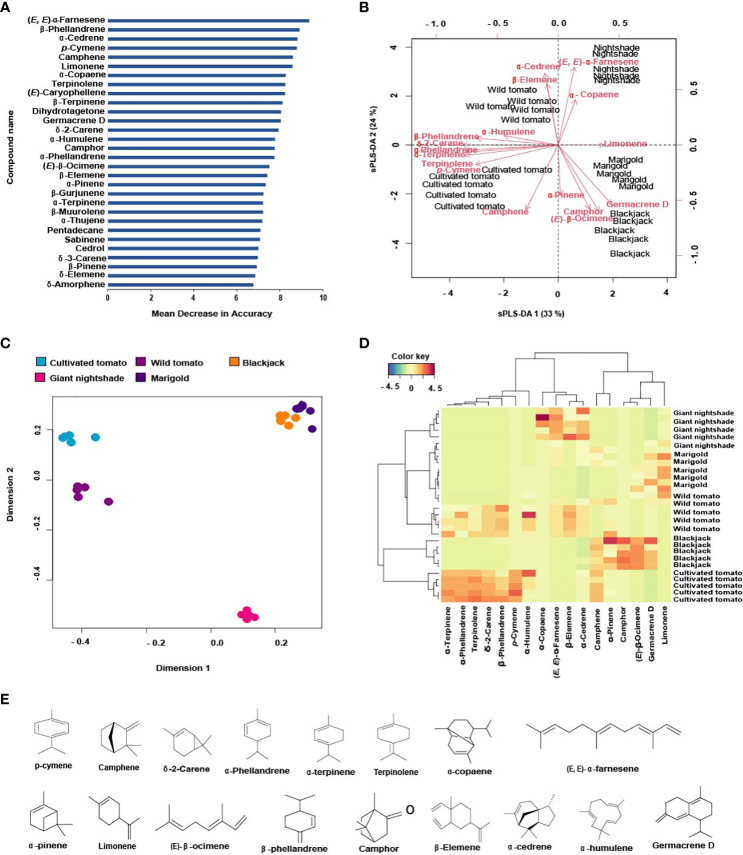
Determination of the most discriminating volatiles and their correlation with host and non-host plants of Tuta absoluta: **(A)** The 30 volatiles that best-distinguished host and non-host plants based on the mean decrease in accuracy of the random forest analysis, **(B)** Multidimensional scaling (MDS) plot showing the distribution of host and non-host plants volatile profiles, **(C)** sPLS-DA biplot showing the correlation of the top discriminating volatiles (R2X = 0.57, R2Y = 0.74, Q2 = 0.58), **(D)** Clustering heatmap showing the abundance of the top discriminating volatiles across replicates of the host and non-host plants, and **(E)** Chemical structures of the top discriminating volatiles.

The response of gravid females of *T. absoluta* and adults of its mirid predator to synthetic standards of the most discriminating compounds associated with the host and non-host plant volatiles identified using the multivariate random forest (RF) analysis were tested in the Y-tube olfactometer as previously described. Of the 17 most discriminating compounds identified (**Figure 1E**), camphene, δ-2-carene, α-phellandrene, α-terpinene, *p*-cymene, terpinolene were associated with cultivated tomato, whereas α-copaene, and (*E*, *E*)-α-farnesene were associated with giant nightshade. Additionally, limonene was associated with marigold, while α-pinene, (*E*)-β-ocimene, camphor, germacrene D were associated with blackjack, and β-phellandrene, β-elemene, α-cedrene, α-humulene were associated with wild tomato (**Table 1** and **Figure 1C**). Blends were formulated to simulate the amounts corresponding to the putative natural concentration detected in the associated plants (**Tables 1**, **2**). Host and non-host plant discriminating volatile blends were tested at three different concentrations, including the standard concentration based on the corresponding source amount detected in the volatiles of the associated plants, double, and half the putative natural concentration (**Table 2** and **Supplementary Table 2**). However, the discriminating compounds α-copaene, camphor, and germacrene D were not included in the blends tested due to their commercial unavailability. Dichloromethane (DCM) was used to dilute the compounds, and a 10 μL aliquot of the test solution was loaded onto a filter paper (2 × 2 cm) and tested against a filter paper loaded with 10 μL solvent control. The impregnated filter papers were air-dried at room temperature for 30 s before placing them into the ends of the olfactometer arms and were changed for every insect tested. Sixty insects were tested per choice test.

**Table 1:** Sources of most discriminating compounds in intact plant volatiles selected for formulating synthetic blends.

Compounds	Cultivated tomato (ng)	Wild tomato (ng)	Giant nightshade (ng)	Marigold(ng)	Blackjack (ng)
Camphene	2.8	–	–	–	–
δ-2-Carene	156.4	–	–	–	–
α-Phellandrene	120.1	–	–	–	–
α-Terpinene	45.7	–	–	–	–
*p*-Cymene	147.1	–	–	–	–
Terpinolene	15.8	–	–	–	–
(*E*, *E*)-α-farnesene	–	–	13.1	–	–
α-pinene	–	–	–	–	98.7
Limonene	–	–	–	26.7	–
β-phellandrene	–	407.3	–	–	–
(*E*)-β-ocimene	–	–	–	–	2.3
β-elemene	–	12.5	–	–	–
α-cedrene	–	4.6	–	–	–
α-humulene	–	17.6	–	–	–

(-) means not selected from.

**Table 2:** Mean amount (ng plant^−1^ h^−1^) of the released volatiles in the headspace of host and non-host plants of *Tuta absoluta*.

Peak No.	R.T (min)	R.I (Calc)	R.I(Lit)	Compound	Chemical class	Mean amount detected (ng plant^−1 ^h^−1^) ± SEM					P-value
						Cultivated tomato	Wild tomato	Nightshade	Marigold	Blackjack	
1	6.49	804	805 ^A^	Hexanal^ɸ^	Aldehyde	1.05	nd	nd	nd	nd	
2	9.17	907	906 ^B^	α-Thujene^ɸ^	Monoterpene	nd	1.60	nd	nd	nd	
3	9.81	934	936 ^C^	α-Pinene ^ɸ^	Monoterpene	28.24 ± 0.53 ^c^	53.18 ± 5.05 ^b^	0.67 ± 0.24 ^d^	0.55 ± 0.12 ^d^	98.71 ± 38.41 ^a^	**0.001**
4	10.09	947	946 ^D^	Camphene ^ɸ^	Monoterpene	2.75 ± 0.23 ^a^	nd	nd	nd	2.57 ± 0.16 ^a^	0.522
5	10.52	961	956 ^E^	*o*-Cymene ^ɸ^	Monoterpene	6.83 ± 2.83 ^a^	26.90 ± 10.61 ^a^	nd	nd	nd	0.119
6	10.65	971	969 ^D^	Sabinene ^ɸ^	Monoterpene	nd	2.89 ± 1.59 ^a^	nd	4.89 ± 0.82 ^a^	2.57 ± 1.13 ^a^	0.299
7	10.69	976	978 ^C^	β-Pinene ^ɸ^	Monoterpene	5.44 ± 1.48 ^ab^	2.81 ± 1.26 ^bc^	0.06 ± 0.01 ^c^	0.37 ± 0.13 ^c^	7.03 ± 2.61 ^a^	**0.001**
8	10.79	980	983 ^C^	(*E*)-isolimonene	Monoterpene	2.86 ± 0.10 ^a^	2.05 ± 1.06 ^a^	nd	nd	nd	0.487
9	10.96	989	993 ^C^	Myrcene ^ɸ^	Monoterpene	5.35 ± 0.28 ^a^	4.56 ± 1.47 ^a^	nd	nd	2.28 ± 0.66 ^a^	0.098
10	11.18	999	1002 ^C^	δ-2-Carene ^ɸ^	Monoterpene	156.38 ± 12.65 ^a^	113.84 ± 1.91 ^b^	nd	nd	nd	**0.027**
11	11.23	1002	1002 ^F^	α-Phellandrene ^ɸ^	Monoterpene	120.12 ± 4.47 ^a^	58.70 ± 18.43 ^a^	nd	nd	0.46 ± 0.13 ^c^	**0.008**
12	11.36	1009	1014 ^G^	δ-3-Carene ^ɸ^	Monoterpene	4.61 ± 0.37 ^ab^	4.69 ± 1.25 ^a^	nd	nd	0.25 ± 0.05 ^b^	**0.008**
13	11.53	1018	1014 ^D^	α-Terpinene ^ɸ^	Monoterpene	45.65 ± 1.37 ^a^	21.70 ± 7.11 ^b^	nd	nd	nd	**0.027**
14	11.65	1025	1020 ^F^	*p*-Cymene ^ɸ^	Monoterpene	147.10 ± 12.70 ^a^	0.95 ± 0.02 ^b^	nd	0.40 ± 0.02 ^c^	nd	**0.008**
15	11.72	1029	1024 ^D^	Limonene ^ɸ^	Monoterpene	nd	nd	2.94 ± 0.54 ^b^	26.69 ± 3.0 ^a^	nd	**0.001**
16	11.81	1034	1034 ^C^	β- Phellandrene ^ɸ^	Monoterpene	330.56 ± 82.02 ^a^	407.33 ± 38.07 ^a^	nd	nd	12.60 ± 2.78 ^b^	**0.008**
17	11.89	1039	1032 ^D^	(*Z*)-β-Ocimene ^ɸ^	Monoterpene	nd	nd	nd	11.30	nd	
18	12.08	1049	1044 ^D^	(*E*)-β-Ocimene ^ɸ^	Monoterpene	nd	nd	nd	0.65 ± 0.21 ^b^	2.32 ± 0.04 ^a^	**0.001**
19	12.14	1049	1046 ^D^	Dihydrotagetone ^ɸ^	Ketone	nd	nd	nd	27.24	nd	
20	12.31	1059	1054 ^D^	β-Terpinene ^ɸ^	Monoterpene	9.73 ± 0.85 ^a^	4.99 ± 2.34 ^a^	nd	nd	nd	0.093
21	12.70	1079	1072 ^B^	Terpinolene ^ɸ^	Monoterpene	15.80 ± 0.90 ^a^	5.89 ± 1.71 ^b^	nd	nd	nd	**0.001**
22	13.45	1128	1121 ^C^	Allo-ocimene	Monoterpene	nd	nd	nd	2.38	nd	
23	13.71	1143	1139 ^D^	(*E*)-Tagetone	Ketone	nd	nd	nd	8.66	nd	
24	13.76	1148	1141 ^D^	Camphor	Monoterpene	nd	nd	nd	nd	0.39	
25	13.86	1154	1148 ^D^	(*Z*)-Tagetone	Ketone	nd	nd	nd	34.04	nd	
26	14.76	1213	1209 ^A^	Decanal ^ɸ^	Aldehyde	nd	3.85 ± 0.19 ^a^	3.03 ± 0.04 ^b^	nd	nd	**0.010**
27	15.09	1239	1235 ^D^	(*E*)-Ocimenone	Ketone	nd	nd	nd	4.05	nd	
28	15.21	1248	1244 ^H^	(*Z*)-Ocimenone	Ketone	nd	nd	nd	64.48	nd	
29	15.23	1250	1251 ^I^	Piperitenone	Ketone	nd	nd	nd	33.02	nd	
30	15.48	1263	1258 ^J^	Caprolactam	Amide	nd	nd	nd	nd	62.80	
31	16.02	1309	1300 ^B^	Tridecane ^ɸ^	Alkane	nd	5.31 ± 0.28 ^a^	1.0 ± 0.10 ^b^	nd	nd	**0.001**
32	16.62	1313	1315 ^B^	δ-Elemene ^ɸ^	Sesquiterpene	7.31 ± 0.67 ^a^	14.33 ± 5.21 ^a^	nd	nd	0.28 ± 0.02 ^b^	**0.008**
33	17.09	1348	1341 ^I^	Hexyl butanoate	Ester	nd	nd	nd	nd	7.16	
34	17.13	1349	1345 ^D^	β-Cubebene	Sesquiterpene	nd	nd	nd	nd	0.50	
35	17.17	1353	1348 ^G^	α-Copaene	Sesquiterpene	nd	nd	12.41	nd	nd	
36	17.26	1371	1374 ^J^	Modheph-2-ene	Sesquiterpene	nd	nd	nd	6.83	nd	
37	17.35	1403	1400 ^C^	β-Elemene ^ɸ^	Sesquiterpene	nd	12.51 ± 0.75 ^a^	9.44 ± 3.40 ^a^	nd	nd	0.424
38	17.55	1407	1406 ^J^	Sesquithujene	Sesquiterpene	nd	nd	4.37	nd	nd	
39	17.62	1409	1409 ^B^	Allo-aromadendrene	Sesquiterpene	nd	nd	0.16	nd	nd	
40	17.71	1411	1410 ^C^	α-Cedrene ^ɸ^	Sesquiterpene	1.53 ± 0.16 ^b^	4.58 ± 0.85 ^a^	3.63 ± 0.05 ^a^	nd	0.23 ± 0.03 ^b^	**<0.001**
41	17.78	1413	1417 ^D^	(*E*)-Caryophyllene ^ɸ^	Sesquiterpene	4.53 ± 0.52 ^b^	89.29 ± 38.77 ^a^	1.08 ± 0.08 ^b^	6.15 ± 2.58 ^b^	8.44 ± 2.03 ^b^	**0.001**
42	17.89	1421	1430 ^D^	β-Copaene	Sesquiterpene	nd	nd	nd	nd	1.19	
43	17.91	1423	1431 ^B^	(*E*)-α-Bergamotene	Sesquiterpene	nd	nd	14.37	nd	nd	
44	17.99	1429	1438 ^B^	β-Gurjunene	Sesquiterpene	nd	nd	nd	nd	0.88	
45	18.22	1448	1452 ^D^	α-Humulene ^ɸ^	Sesquiterpene	13.36 ± 6.06 ^ab^	17.57 ± 6.52 ^a^	nd	2.74 ± 0.68 ^b^	0.26 ± 0.03 ^c^	**0.008**
46	18.45	1476	1478 ^D^	β-Muurolene	Sesquiterpene	nd	nd	1.01	nd	nd	
47	18.54	1487	1480 ^E^	Germacrene D	Sesquiterpene	nd	2.53 ± 0.03 ^b^	nd	5.26 ± 1.56 ^b^	8.89 ± 1.69 ^a^	**0.004**
48	18.63	1498	1500 ^J^	Pentadecane ^ɸ^	Alkane	1.82 ± 0.57 ^b^	8.49 ± 0.83 ^a^	8.43 ± 0.22 ^a^	nd	nd	**0.008**
49	18.75	1508	1500 ^D^	Bicyclogermacrene	Sesquiterpene	nd	nd	nd	4.84	nd	
50	18.76	1509	1509 ^J^	β-Curcumene	Sesquiterpene	nd	nd	nd	0.04	nd	
51	18.77	1510	1509 ^I^	(*E*, *E*)-α-farnesene ^ɸ^	Sesquiterpene	nd	7.75 ± 0.20 ^b^	13.05 ± 0.55 ^a^	nd	nd	**0.001**
52	18.81	1513		Unidentified		nd	nd	nd	nd	83.21	
53	19.04	1523	1515 ^B^	δ-Amorphene	Sesquiterpene	nd	nd	nd	0.48	nd	
54	19.49	1571	1576 ^F^	(*E*)- Farnesol	Sesquiterpene	nd	nd	11.64	nd	nd	
55	19.92	1610	1622 ^A^	Cedrol ^ɸ^	Alcohol	nd	5.03	nd	nd	nd	
56	19.98	1611	1611 ^B^	Hexadecane ^ɸ^	Alkane	2.27 ± 0.78 ^b^	11.12 ± 1.59 ^a^	4.52 ± 0.18 ^b^	nd	nd	**<0.001**

R.T (min) = Retention time in minutes, R.I (Calc) = Retention index calculated based on the C_8_- C_31_ n- alkanes of HP- 5 MS column, R.I (Lit) = Retention index obtained from literature: A (Hassaballa et al., 2021), B (Antwi-Agyakwa et al., 2021), C (Ayelo et al., 2021), D (Matu et al., 2021), E (Njuguna et al., 2018), F (Adams2, 1995), G (Kihika et al., 2020), H (Diabate et al., 2019), I (Silva et al., 2017), J (NIST, 2008), P-value = Probability value of the non-parametric test for comparing the amounts of volatiles released by cultivated tomato, wild tomato, giant nightshade, marigold, and blackjack where significant values are in bold and means with different letters are significantly different at 5% probability level (P ≤ 0.05), nd = not detected, the symbol (^ɸ^) = Compounds confirmed with authentic standards.

To prepare the synthetic blends, a stock solution containing 1mg/mL of each compound was formulated individually by diluting 1mg (0.001 g) of each compound with 1mL (1000 µL) of dichloromethane (DCM) solvent in a 1mL GC vial. The concentration (working solution) of each compound selected for the behavioral assay as detected in the corresponding plants was then formulated from the stock solution of each compound separately. Equal volumes of the working solution of each compound containing the corresponding concentrations detected in our plants were then pipetted individually and combined into a 1mL GC vial. This procedure was followed to formulate all the blends used in our study.

### 2.10 Statistical analysis

Data analyses were done using R software 64 (R version 4.2.0) and the R Studio graphical user interface (R Core Team, 2018). The frequency count from the Y-tube olfactometer bioassays were subjected to Chi-square (χ^2^) goodness-of-fit analysis. The number of *T. absoluta* females or adults of its mirid predator responding to each treatment in each experiment was expressed as a percentage using the formula [(n/N) × 100], where n is the number of *T. absoluta* or adults of *N. tenuis* responding to a given treatment, while N is the total number of *T. absoluta* or the mirid predator responding. Non-responding *T. absoluta* or *N. tenuis* were not included in the analysis.

To analyze the differences in the emission of volatile organic compounds between the host and non-host plants of *T. absoluta*, the normality of the data was first tested using the Shapiro–Wilk’s test and the data was found to be not normally distributed, hence, a non-parametric analysis was done using Kruskal–Wallis test and an unpaired T-test. Means were separated (*post hoc* test) using Mann– Whitney–Wilcoxon test and Tukey’s honest significant difference (HSD) where applicable. The same non-parametric and *post-hoc* tests were used to analyze the distribution of the compounds in the attractive and repellent mono- and sesqui-terpene blends across the various host and non-host plants. All the analyses were done at 5% probability level (*P* ≤ 0.05).

Using the machine learning algorithm random forest (RF), the volatiles that best distinguish the host and non-host plants were selected based on the mean decrease in accuracy (MDA) (Ayelo et al., 2021). Compounds were grouped into random groups and the most consistent variables among the groups were selected as the most important. This was done using the random forest ‘importance’ function and the out-of-bag error (OOB error) which predicts the accuracy of the analysis (Ranganathan and Borges, 2010). Moreover, a multidimensional scaling analysis (MDS) was done to visualize the similarity of the volatile profiles of the host and non-host plants of *T. absoluta* using the random forest ‘MDSplot’ function (Liaw and Wiener, 2002).

A sparse partial least square discriminant analysis (sPLS-DA) biplot was used to visualize how the most discriminating volatiles correlate with each host and non-host plant using the mixOmics package (Lê Cao et al., 2011). With the aid of the function ‘perf’ sPLS-DA parameters (R2X, R2Y and Q2) and ‘leave-one-group-out’ cross-validation method, the sPLS-DA model was validated (Rohart et al., 2017). R2X explained the variation in the most discriminating VOC’s, R2Y the variation in the groups (plants), and Q2 the average predictive capability of the model. To visualize the variations in the emission of the most discriminating volatiles across replicates profiles of the host and non-host plants, a clustering heatmap was generated using the function ‘cim’ in the mixOmics package (Rohart et al., 2017).

## 3 Results

### 3.1 Molecular identity of the mirid predator

Molecular characterization of the predator targeting the 28S rDNA large ribosomal domain 2 (D2) gene region identified the predator up to the family level Miridae. Due to no amplification observed using the universal Folmer primers (HCO/LCO) (Folmer et al., 1994), the complete mitochondrial COI gene was obtained from WGS, and this resolved the identity of the predator up to species level. A BLAST search of the extracted COI gene generated from this study (Accession no. OP781962) had a 97.53% similarity with *N. tenuis* (JQ806057.1), hence the predator was conclusively determined to be *N. tenuis* Reuter.

### 3.2 Behavioral response of *T. absoluta* to host and non-host plant volatiles

**Figure 2:**
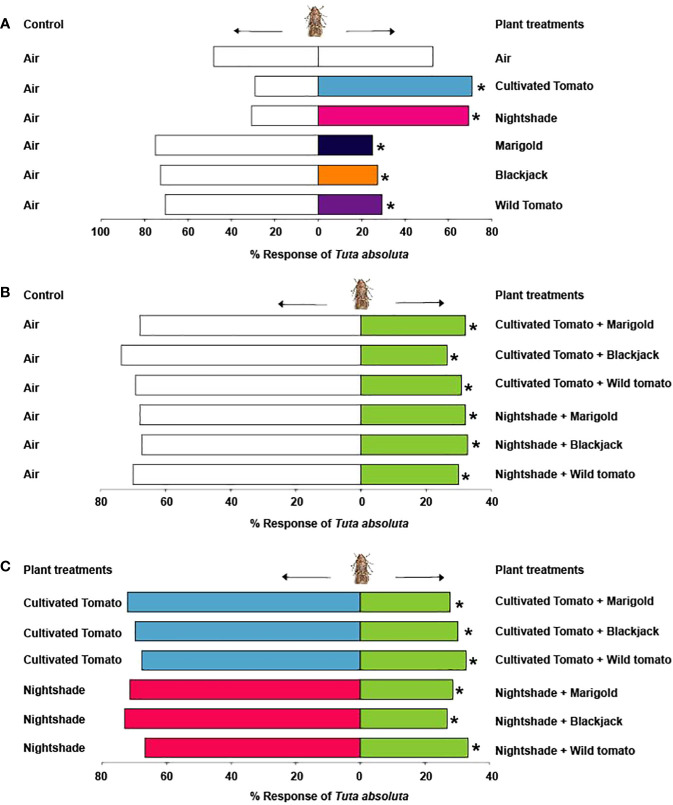
Behavioural response of Tuta absoluta to **(A)** Solanaceae and Asteraceae plants relative to air control, **(B)** Combination of Solanaceae and Asteraceae plants relative to air control, **(C)** Combination of Solanaceae and Asteraceae plants relative to the Solanaceae plants. Asterisks (*) indicate significant differences (Chi-square test: P = 0.05).

Tests with empty roasting bags alone showed no significant difference in the response of *T. absoluta* females to the two arms (χ^2^ = 0.02, df = 1, *P* = 0.89). *Tuta absoluta* females were significantly attracted to volatiles of cultivated tomato (70.9%, χ^2^ = 8.80, df = 1, *P* = 0.003), nightshade (69.2%, χ^2^ = 6.94, df = 1, *P* = 0.008), but significantly avoided volatiles from the non-host Asteraceae plants marigold (75.0%, χ^2^ = 12.02, df = 1, *P* = 0.001), blackjack (72.7%, χ^2^ = 10.47, df = 1, *P* = 0.001), and wild tomato (70.6%, χ^2^ = 7.84, df = 1, *P* = 0.005) relative to the air controls (**Figure 2A**). When presented with a combination of volatiles from cultivated tomato and the non-host plants against clean air, females significantly avoided the combined volatiles of cultivated tomato and marigold (67.9%, χ^2^ = 6.45, df = 1, *P* = 0.01), cultivated tomato and blackjack (73.6%, χ^2^ = 10.87, df = 1, *P* = 0.001), and cultivated tomato and wild tomato (69.2%, χ^2^ = 6.94, df = 1, *P* = 0.01) (**Figure 2B**). Likewise, they avoided the combined volatiles of nightshade and marigold (67.9%, χ^2^ = 6.45, df = 1, *P* = 0.01), nightshade and blackjack (67.3%, χ^2^ = 5.89, df = 1, *P* = 0.02), and nightshade and wild tomato (70.0%, χ^2^ = 7.22, df = 1, *P* = 0.007) relative to the air controls (**Figure 2B**). Moreover, females significantly avoided a combination of cultivated tomato and marigold (72.2%, χ^2^ = 9.13, df = 1, *P* = 0.002), cultivated tomato and blackjack (69.8%, χ^2^ = 7.55, df = 1, *P* = 0.006), and cultivated tomato and wild tomato (67.3%, χ^2^ = 5.89, df = 1, *P* = 0.02) relative to cultivated tomato (**Figure 2C**). Likewise, they significantly avoided a combination of nightshade and marigold (71.4%, χ^2^ = 9.45, df = 1, *P* = 0.002), nightshade and blackjack (73.1%, χ^2^ = 10.17, df = 1, *P* = 0.001), and nightshade and wild tomato (66.7%, χ^2^ = 5.02, df = 1, *P* = 0.03) relative to nightshade (**Figure 2C**).

### 3.3 Coupled gas chromatography-mass spectrometric identification of host and non-host plant volatiles

**Figure 3:**
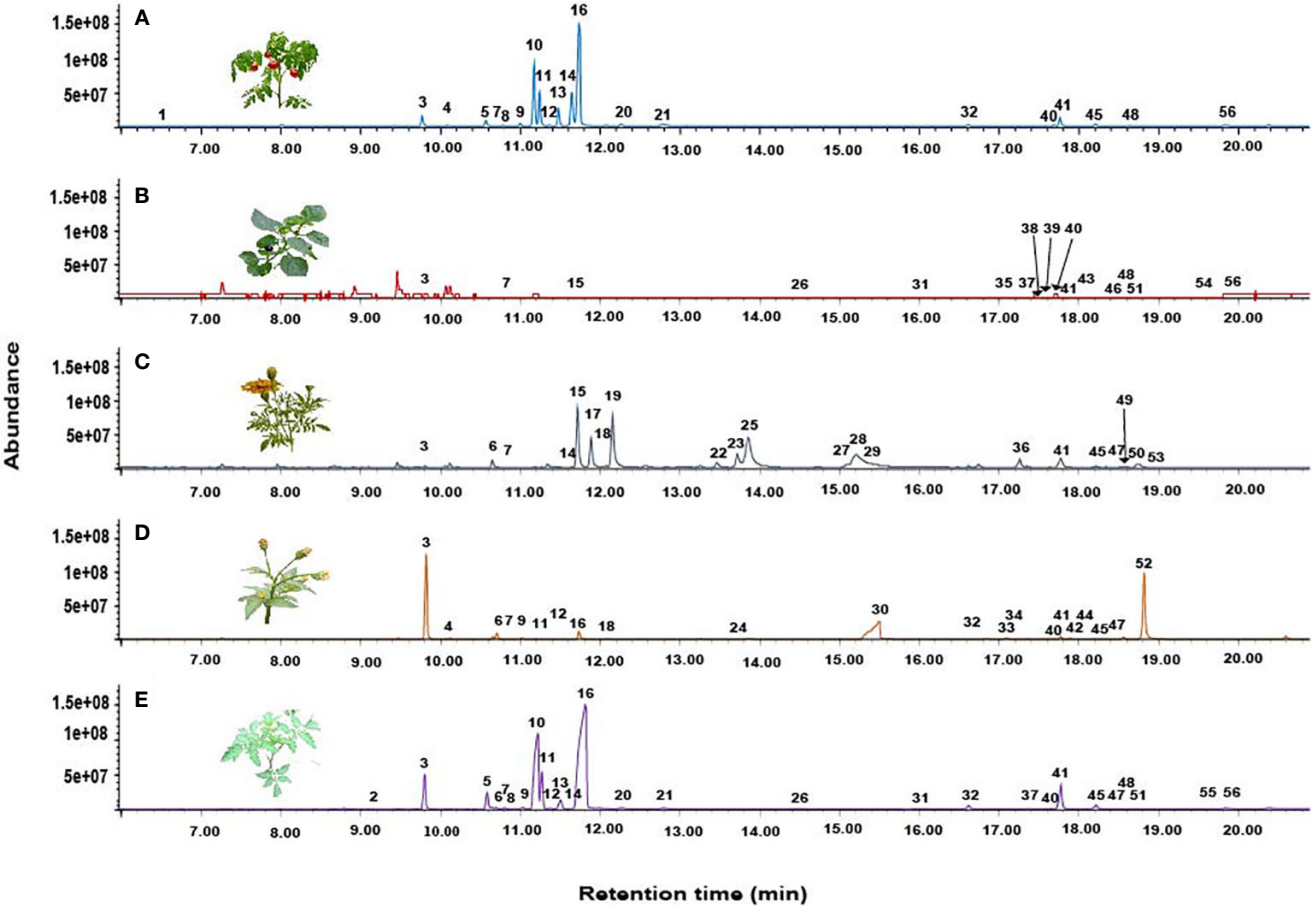
Representative total ion chromatograms (TICs) showing the headspace volatile profiles **(A)** Cultivated tomato, **(B)** Giant nightshade **(C)** Marigold, **(D)** Blackjack, and **(E)** Wild tomato.

GC-MS analysis identified 56 compounds in the volatiles of host and non-host plants, belonging to eight chemical classes namely, monoterpenes, sesquiterpenes, ketones, aldehydes, amides, alkanes, esters, and alcohols. These volatiles varied quantitatively and qualitatively (**Table 2**). A total of 21 compounds were detected in the volatile profile of cultivated tomato, wild tomato (27), nightshade (17), marigold (21), and blackjack (21) (**Table 2** and **Figure 3**). The monoterpenes β-phellandrene (36.2%) and δ-2-carene (17.5%) were the most abundant compounds detected in the volatiles of cultivated tomato, whereas for wild tomato volatiles the monoterpene β-phellandrene (45.5%) and the sesquiterpene (*E*)-caryophyllene (10.1%) were the most dominant compounds. The sesquiterpenes (*E*, *E*)-α-farnesene (14.8%) and α-copaene (14.0%), and the ketones (*Z*)-ocimenone (28.2%) and (*Z*)-tagetone (14.9%) dominated the volatile profiles of giant nightshade and marigold, respectively. The abundant compounds in blackjack volatiles were the monoterpene α-pinene (32.7%) and an unidentified compound (27.2%) (**Table 2**). In all the assays, the non-responding females were less than 15%.

### 3.4 Identification and determination of discriminant volatiles from host and non-host plants

Using the machine learning algorithm random forest (RF), 30 volatile organic compounds (VOC_S_) were identified as the most discriminating compounds among the host and non-host plants with (*E*, *E*)-α-farnesene being the most discriminating compound (**Figure 1A**). The RF algorithm classified the variables or compounds based on ‘mean decrease in accuracy’ (MDA) in order of importance. The accuracy of the classification [out-of-bag error (OOB error)] was 100%. Based on the MDS the plants were grouped into four clusters based on their volatile profiles. Cultivated tomato (cluster one), wild tomato (cluster two), giant nightshade (cluster three), and cluster four includes marigold and blackjack (**Figure 1B**). Further sparse partial least square discriminant analysis (sPLS-DA), selected 17 volatile organic compounds (**Figure 1E**) out of the 30 as the best distinguished compounds namely; camphene, δ-2-carene, α-phellandrene, α-terpinene, *p*-cymene, terpinolene, α-copaene, (*E*, *E*)-α-farnesene (from host plants) and α-pinene, limonene, β-phellandrene, (*E*)-β-ocimene, camphor, β-elemene, α-cedrene, α-humulene, and germacrene D (from non-host plants). The quality parameters of the sPLS-DA model (R2X = 0.57, R2Y = 0.74, Q2 = 0.58) show that appropriate classification was achieved (**Figure 1C**). The difference between R2Y and Q2 was less than 0.2, and the Q2 value was greater than 50%, indicating an excellent predictive capability. The total variations in the sPLS-DA (57%) were explained by the first two dimensions, with dimension 1 and 2 accounting for 33% and 24%, respectively. In dimension 1, α-terpinene, *p*-cymene, α-phellandrene, terpinolene, δ-2-carene correlated with cultivated tomato, β-phellandrene and α-humulene correlated with wild tomato, limonene correlated with marigold, and (*E*)-β-ocimene and germacrene D correlated with blackjack (**Figure 1C**). In dimension 2, camphene correlated with cultivated tomato, β-elemene and α-cedrene correlated with wild tomato, α-copaene, (*E*, *E*)-α-farnesene correlated with nightshade, α-pinene and camphor also correlated with blackjack (**Figure 1C**). Heatmap clustering showed that there were quantitative and qualitative differences among the 17 best distinguished VOCs across the various replicates of the host and non-host plants (**Figure 1D**). Among the top eight discriminating VOCs distinguishing the host plants, seven compounds that were commercially available and tested in various blends in a Y-tube olfactometer dual-choice assay in our study were camphene, δ-2-carene, α-phellandrene, α-terpinene, *p*-cymene, terpinolene, (*E*, *E*)-α-farnesene, while the seven compounds from the non-host plants were α-pinene, limonene, β-phellandrene, (*E*)-β-ocimene, β-elemene, α-cedrene, α-humulene out of the nine discriminating non-host plant compounds.

### 3.5 Response of *T. absoluta* to synthetic blends of host and non-host plant volatiles

**Figure 4:**
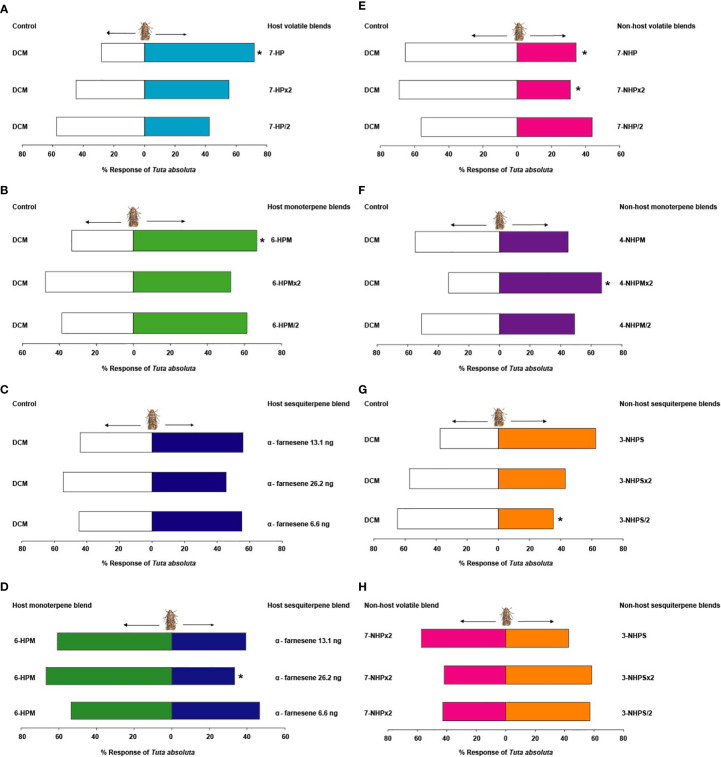
Behavioural response of *Tuta absoluta* females to the most discriminating synthetic **(A)** seven-component host plant volatile blend, **(B)** six-component host plant monoterpene blend, **(C)** host plant sesquiterpene (E, E)-α-farnesene, **(D)** most attractive six-component blend of the host plant monoterpenes relative to host plant sesquiterpenes, **(E)** seven-component non-host plant volatile blend, **(F)** four-component non-host plant monoterpene blend, **(G)** three-component non-host plant sesquiterpene blend, and **(H)** most effective seven-component blend of the non-host plants relative to non-host plant sesquiterpenes. Asterisk (*) indicates significant differences (Chi-square test: *P* = 0.05).

Female moths were significantly attracted to the seven-component (camphene, δ-2-carene, α-phellandrene, α-terpinene, *p*-cymene, terpinolene, (*E*, *E*)-α-farnesene) blend (7-HP) formulated from the host plant discriminating volatiles (71.9%, χ^2^ = 10.11, df = 1, *P* = 0.001), but no significant behavioural response was observed when the concentration was doubled (7-HP×2) (χ^2^ = 0.43, df = 1, *P* = 0.51), or halved (7-HP/2) (χ^2^ = 1.08, df = 1, *P* = 0.29) relative to the solvent control (**Figure 4A**). Likewise, adult female moths were significantly attracted to the six-component (camphene, δ-2-carene, α-phellandrene, α-terpinene, *p*-cymene, terpinolene) blend of the host plant monoterpenes (6-HPM) (66.7%, χ^2^ = 5.68, df = 1, *P* = 0.02), but showed no preference to double (6-HPM×2) (χ^2^ = 0.07, df = 1, *P* = 0.79), and half the concentration (6-HPM/2) (χ^2^ = 2.53, df = 1, *P* = 0.11) relative to the control (**Figure 4B**). Contrastingly, the sesquiterpene (*E*, *E*)-α-farnesene did not elicit any significant behavioral response in females of *T. absoluta* at all the concentrations tested; putative natural concentration (i.e. 13.1 ng (*E*, *E*)-α-farnesene) (χ^2^ = 0.84, df = 1, *P* = 0.36), double (i.e. 26.2 ng (*E*, *E*)-α-farnesene) (χ^2^ = 0.29, df = 1, *P* = 0.59), and half the putative natural concentration (i.e. 6.6 ng (*E*, *E*)-α-farnesene) (χ^2^ = 0.43, df = 1, *P* = 0.51) relative to the control (**Figure 4C**). Interestingly, when *T. absoluta* was presented with the attractive host plant monoterpene blend (6-HPM) and the host plant sesquiterpenes, the adult females significantly preferred the attractive host plant monoterpenes (6-HPM) (60.7%, χ^2^ = 5.68, df = 1, *P* = 0.02) over the host plant sesquiterpenes at the putative natural concentration (i.e. 13.1 ng (*E*, *E*)-α-farnesene). No significant response was observed at double (i.e. 26.2 ng (*E*, *E*)-α-farnesene) (χ^2^ = 2.16, df = 1, *P* = 0.14) and half the putative natural concentration (i.e. 6.6 ng (*E*, *E*)-α-farnesene) (χ^2^ = 0.15, df = 1, *P* = 0.69) (**Figure 4D**).

The seven-component (limonene, α-pinene, (*E*)-β-ocimene, β-phellandrene, β-elemene, α-cedrene, α-humulene) blend of the most discriminating non-host plant volatiles significantly repelled *T. absoluta* females when tested at the putative natural concentration (7-NHP) (65.5%, χ^2^ = 5.68, df = 1, *P* = 0.02) and double the concentration (7-NHP×2) (68.9%, χ^2^ = 7.60, df = 1, *P* = 0.006) relative to the solvent control. No significant behavioural response was observed when half the putative natural concentration was tested (7-NHP/2) (χ^2^ = 0.63, df = 1, *P* = 0.43) (**Figure 4E**). Surprisingly, *T. absoluta* females were significantly attracted to a four-component (limonene, α-pinene, (*E*)-β-ocimene, β-phellandrene) blend of the non-host plant monoterpenes containing double the putative natural concentration (4-NHPM×2) (66.7%, χ^2^ = 5.68, df = 1, *P* = 0.02), whereas no significant response was observed to the putative natural (4-NHPM) (χ^2^ = 0.43, df = 1, *P* = 0.51) and half this concentration (4-NHPM/2) (χ^2^ = 0, df = 1, *P* = 1) relative to the control (**Figure 4F**). Interestingly, the non-host plant three-component (β-elemene, α-cedrene, α-humulene) sesquiterpene blend significantly repelled females of *T. absoluta* at half the putative natural concentration (3-NHPS/2) (64.8%, χ^2^ = 4.17, df = 1, *P* = 0.04). No significant response was observed at the putative natural concentration (3-NHPS) (χ^2^ = 3.02, df = 1, *P* = 0.08) and double this concentration (3-NHPS×2) (χ^2^ = 0.86, df = 1, *P* = 0.34) relative to the control (**Figure 4G**). When presented with the most effective non-host plant seven-component blend (7-NHP×2) and the three-component non-host plant sesquiterpene blends individually, the female moths did not show any preference to any of these blends at all the concentrations tested; putative natural concentration (3-NHPS) (χ^2^ = 0.91, df = 1, *P* = 0.34), double (3-NHPS×2) (χ^2^ = 1.16, df = 1, *P* = 0.28), or half the putative natural concentration (3-NHPS/2) (χ^2^ = 0.86, df = 1, *P* = 0.35) (**Figure 4H**).

### 3.6 Response of *N. tenuis* to host monoterpene and non-host sesquiterpene synthetic blends

**Figure 5:**
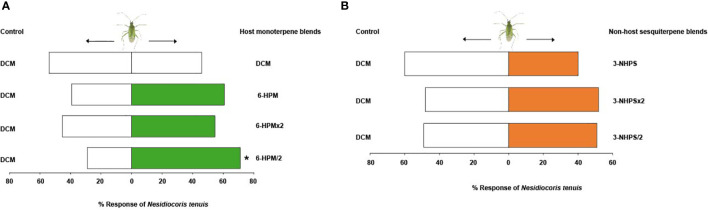
Behavioural response of adults of Nesidiocoris tenuis to the most discriminating synthetic **(A)** six-component host plant monoterpene blend and **(B)** three-component non-host plant sesquiterpene blend. Asterisk (*) indicates significant differences (Chi-square test: P = 0.05).

Tests with the solvent control (DCM) alone showed no significant difference in the response of adults of *N. tenuis* to the two arms of the olfactometer (χ^2^ = 0.18, df = 1, *P* = 0.67). Adults of *N. tenuis* were significantly attracted to half the putative natural concentration of the host plant six-component monoterpene blend (6-HPM/2) (71.2%, χ^2^ = 8.48, df = 1, *P* = 0.004), but were indifferent to the putative natural (6-HPM) (χ^2^ = 1.96, df = 1, *P* = 0.16) and double the putative natural concentration (6-HPM×2) (χ^2^ = 0.30, df = 1, *P* = 0.58) relative to the control (**Figure 5A**). Interestingly, the three-component blend of the non-host plant sesquiterpenes did not elicit any significant behavioral response in adults of *N. tenuis* when tested at all concentrations: putative natural (3-NHPS) (χ^2^ = 1.62, df = 1, *P* = 0.20), double (3-NHPS×2) (χ^2^ = 0.02, df = 1, *P* = 0.89), and half the putative natural concentration (3-NHPS/2) (χ^2^ = 0, df = 1, *P* = 1) relative to the control (**Figure 5B**).

### 3.7 Discriminating volatiles reveal the most attractive and repellent host and non-host plants

**Figure 6:**
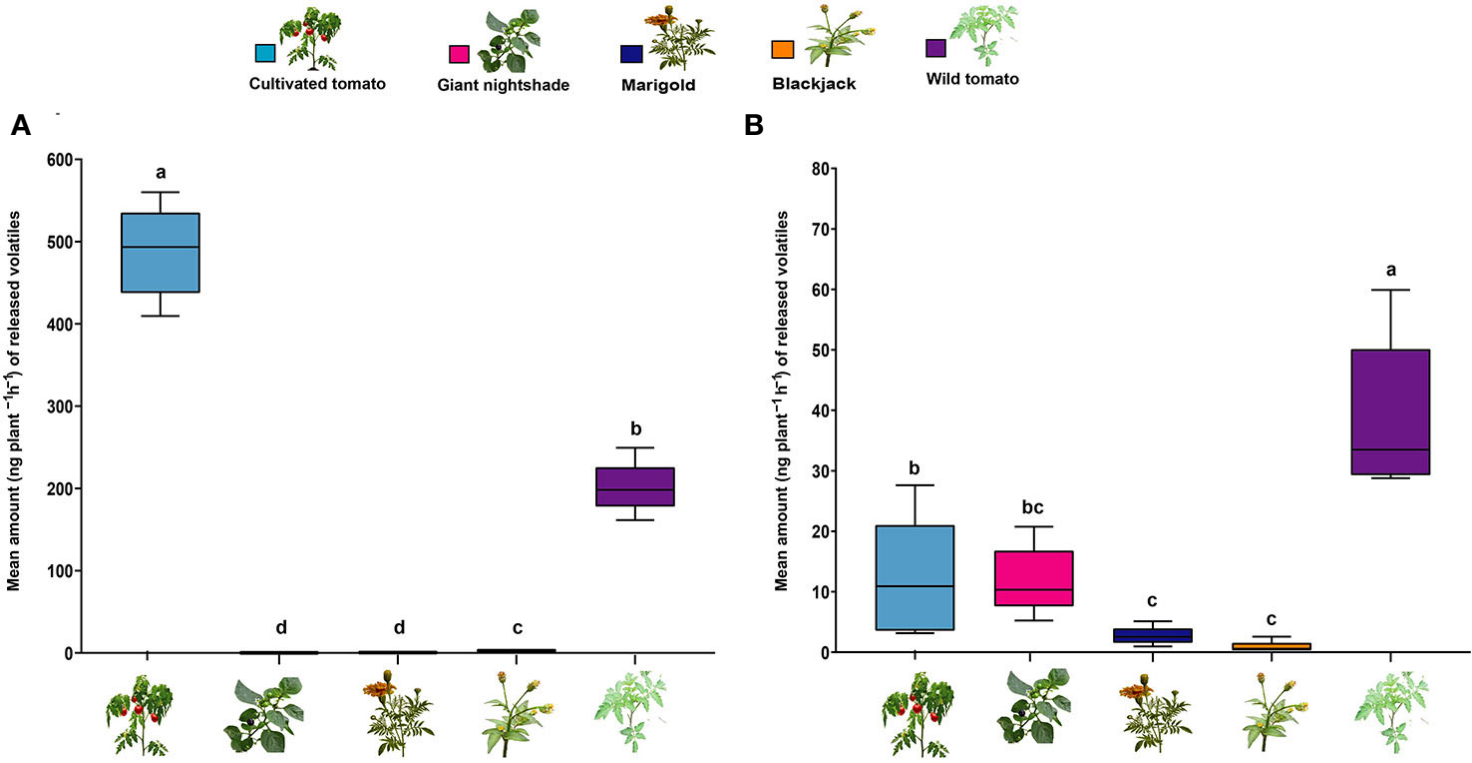
Mean concentration of volatile organic compounds (VOCs) detected from different host and non-plants. Box plot of VOCs from **(A)** most attractive six-component blend of the host plant monoterpenes comprising camphene, δ-2-carene, α-phellandrene, α-terpinene, p-cymene, terpinolene and **(B)** most effective three-component non-host plant sesquiterpene blend comprising β-elemene, α-cedrene, α-humulene. Means with the same letter are not significantly different (Tukey’s HSD test, P ≤ 0.05).

We found that cultivated tomato followed by wild tomato significantly emitted (Kruskal–Wallis test, P = 0.008, df = 20, N = 5) the highest amount of the total attractive host plant monoterpene blend (6-HPM) compared to marigold, blackjack, and nightshade which emitted the least amount. (**Figure 6A**). Cultivated tomato’s release of the monoterpene blend was approximately 3- 1200-fold more than the other plants. On the other hand, using the three-component repellent sesquiterpene blend (3-NHPS/2) (**Figure 6B**), wild tomato significantly emitted the highest amount compared to the other plants (*P* = 0.007, df = 20, N = 5). Except for the non-host plant wild tomato, which released about 3- 69-fold more sesquiterpenes, the levels of this blend released by the host and the other non-host plants were relatively lower and significantly different.

## 4 Discussion

Our results indicate that adult females of *T. absoluta* are attracted to Solanaceae host plant volatiles but avoid host plant volatiles in the presence of the non-host Asteraceae plants marigold, blackjack, and the positive control, wild tomato. These results are consistent with the fact that olfactory cues play an important role in the host finding process of *T. absoluta* (Desneux et al., 2010; Bitew, 2018; Subramani et al., 2021). Our findings corroborate results of a previous study (Miano et al., 2022), which demonstrated the host disruption and masking role of the volatiles of the positive control, wild tomato in the host location process of *T. absoluta* in the presence of the host plant cultivated tomato.

The results of the present study also show that wild tomato volatiles may exert a similar host disruption effect on *T. absoluta* in the presence of giant nightshade which is another Solanaceae host plant. Interestingly, our results also show that volatiles of the non-host Asteraceae plants appear to exert a similar masking effect on host location by *T. absoluta* in the presence of both the two host plants, cultivated tomato and giant nightshade. It has been suggested that the disruption of the host finding process in *T. absoluta* females by the positive control, wild tomato increases with increasing plant age (Rakha et al., 2017). It would be interesting to investigate whether similar age-related host disruption effects occur in the non-host Asteraceae plants. The use of Asteraceae plants as companion crops to disrupt host location in certain insects has been previously reported. For example, in an intercropping system, the Asteraceae plant gallant soldier (*Galinsoga parviflora*) reduced the abundance of *T. absoluta* on tomato (Medeiros et al., 2009). Although host disruption in *T. absoluta* was attributed to *G*. *parviflora* volatiles, the mediating volatiles were not identified. In another study, the essential oils from the Asteraceae plants, *Artemisia absinthium* and *Eupatorium buniifolium* were found to repel two common tomato pests *T. absoluta* and *T. vaporariorum* (Umpiérrez et al., 2012). The oxygenated monoterpenes α- and β-thujone accounted for the repellence of the two tomato pests by *A. absinthium*, whereas the monoterpene hydrocarbon α-pinene and the sesquiterpene hydrocarbon (*E*)-β-guaiene were identified as the repellents for *E. buniifolium*. Additionally, the application of blackjack extract as a biopesticide reduced populations of the aphid *Aphis fabae*, the bean foliage beetle *Ootheca mutabilis*, and the flower beetle *Epicauta albovittata* on cowpea, which translated into a significant reduction in cowpea yield loss (Tembo et al., 2018). The headspace volatiles and essential oil from the flowers of marigold negatively impacted the reproduction and feeding behavior of the green peach aphid (*M. persicae*) and the greenhouse whitefly (*T. vaporariorum*), both of which are above-ground pests of solanaceous plants including pepper and tomato (Tomova et al., 2005; Dardouri et al., 2021; Matu et al., 2021). These previous reports and our findings confirm that phytochemicals of wild tomato and Asteraceae plants interrupt the host finding process of *T. absoluta* females.

Chemical analysis identified terpenes as the dominant compounds in the headspace volatiles of the host and non-host plants. In our olfactometer dose-response bioassays, we found that the six-component monoterpene blend (camphene, δ-2-carene, α-phellandrene, α-terpinene, p-cymene, terpinolene) with the highest amount emitted by cultivated tomato was attractive to *T. absoluta* females. Evidence of the role of monoterpenes in the attraction of insects to host plants has been well documented (Silva et al., 2017; Njuguna et al., 2018; Msisi et al., 2020). For instance, in a laboratory study, α-terpinene, a key shared compound among the three host plants of *T. absoluta* (tomato, aubergine, and sweet pepper) in a five-component blend attracted the females of *T. absoluta* (Msisi et al., 2020). Likewise, the monoterpenes α-phellandrene and *p*-cymene were identified as key constituents of a seven-component blend that attracted the melon fly, *Zeugodacus cucurbitae* (Coquillett), a pest of cucumber and tomato plants (Njuguna et al., 2018).

Our molecular studies identified the mirid predator as *N. tenuis*, a known generalist predator of many insect pests of vegetable crops. In bioassays, whereas the monoterpene blend elicited an attractive response in *T. absoluta* at a higher dose, it was attractive to *N. tenuis* at a relatively lower dose. This suggests that *N. tenuis* is more sensitive to detecting host attractants than its prey. It appears that *N. tenuis* associates the detection of host plant volatiles with the presence of its prey. This agrees with a previous study which demonstrated the attraction of *N. tenuis* to healthy and *T. absoluta* infested host plant volatiles (Lins et al., 2014; Naselli et al., 2017). However, in the absence of the prey, *N. tenuis* also feeds on the host plant. Perhaps *N. tenuis* uses host plant attractants as a habitat selection cue, while it uses visual cues to detect its prey on the host plant. A previous study where the sex pheromones of its prey *T. absoluta* failed to elicit any behavioral response in the adults of *N. tenuis* further strengthen this assertion (Lins et al., 2014). Investigating the cues mediating these interactions would enhance our understanding on how the mirid predator finds its prey. Previously, headspace volatiles of tomato plants dominated by terpenes including the monoterpenes α-terpinene and γ-terpinene have been found to attract the three Neotropical mirid predators *Macrolophus basicornis*, *Campyloneuropsis infumatus* and *Engytatus varians* (Silva et al., 2017). Additionally, *N. tenuis* is attracted to the monoterpenes α-pinene, α-phellandrene, 3-carene, β-phellandrene and β-ocimene (Ayelo et al., 2021). Certain non-host plants have been found to conserve the population of some species of mirids of the genus *Macrolophus* to provide inoculum that augments the natural control of insect pests in adjacent crops (Castañé et al., 2004; Perdikis et al., 2007). For instance, the non-cultivated perennial Asteraceae plant *Dittrichia viscosa* has been reported to conserve the population of the mirid predator *Macrolophus melanotoma* by serving as a shelter and alternate food source (Perdikis et al., 2007). In a greenhouse study, *D. viscosa* was found to enhance the development time, fecundity and the intrinsic rate of population increase of *M. melanotoma* (Perdikis et al., 2007). This aspect also deserves further investigation using the non-host Asteraceae plants tested in the present study for conserving populations of the mirid predator *N. tenuis*.

Interestingly, the amounts of the attractive monoterpene blend in the volatiles of nightshade were relatively low and not significantly different from the amounts detected in the volatiles of the Asteraceae plants, yet the intact nightshade plant was attractive, while the non-host Asteraceae plant volatiles were avoided by the herbivore. These results suggest that the amounts and ratios of attractive compounds and background odor chemicals including sesquiterpenes, ketones, aldehydes, amides, alkanes, esters, and alcohols in both host and non-host plants may all contribute to the overall quality of the signal detected by the herbivore in behavioral response. This is consistent with the fact that the four-component synthetic monoterpene blend identified from the non-host Asteraceae plants attracted *T. absoluta* females, but the intact plants which released a diverse group of volatiles were avoided by females. More research is needed to understand the roles of the other classes of compounds in both the behavior of the herbivore and natural enemy.

Behavioral assays identified the sesquiterpenes β-elemene, α-cedrene, and α-humulene as repellent to the herbivore *T. absoluta*, whereas the mirid predator was indifferent. Although the chemical profiles of cultivated and wild tomato were similar, we found that these sesquiterpenes were most abundant in the volatiles of wild- than cultivated-tomato with relatively low levels detected in nightshade and the non-host Asteraceae plants. These results indicate that the differential levels of sesquiterpenes released by host and non-host plants combined with the background volatiles may account for the variation in the repellent and indifferent responses elicited in the herbivore and predator, respectively. The importance of sesquiterpenes in host plant selection by insect pests has been previously demonstrated (Antonio et al., 2010; Liu et al., 2021; Wang et al., 2021). For example, hybrid tomato cultivars with high acyl sugar and/or high zingiberene (sesquiterpene) allelochemicals reduced the oviposition and leaf damage of *T. absoluta* and other insect pests of tomato than tomato varieties with low acyl sugar and/or high zingiberene (Antonio et al., 2010). An increase in acyl sugar was found to be positively correlated to an increase in the age of wild tomato plants (Rakha et al., 2017), suggesting an increase in resistance of wild tomato to *T. absoluta* with increasing age of the plant. Whether this increase in acyl sugar level may have implication in sesquiterpene levels would require additional research. This could explain the observed repellence of *T. absoluta* by wild tomato in our study. Moreover, the sesquiterpenes β-elemene, germacrene-D, δ-elemene, β-cedrene identified in the essential oil of the leaves of *Zanthoxylum rhoifolium* were found to significantly reduce (as high as 95%) the number of eggs and nymphs of the whitefly*, B. tabaci* (Christofoli et al., 2015). α-Humulene was found to be the most toxic component of the essential oil of the rhizome of the Asian plant species, *Cheilocostus speciosus* against the Old-World bollworm, *Helicoverpa armigera*, a pest of sorghum, legumes, and several horticultural crops (Benelli et al., 2018). Additionally, β-caryophyllene and α-humulene identified in tomato odor were found to negatively affect the performance, choice behavior and feeding of the potato aphid, *Macrosiphum euphorbiae* (Wang et al., 2021).

In summary, the results from our study show that in the tomato-*T. absoluta*- *N. tenuis* tritrophic interaction, monoterpenes released at varying levels from the host plants cultivated tomato and related plant giant nightshade serve as kairomones for *T. absoluta* and *N. tenuis*, whereas sesquiterpenes from the Asteraceae plants marigold, blackjack, and the positive control, wild tomato are allomones for *T. absoluta*. However, our limited results show that the non-host plant repellent volatiles may not influence the behavior of the mirid predator *N. tenuis.* Our findings appear to lay the foundation for intercropping either wild tomato, marigold or blackjack with the host plants cultivated tomato or nightshade for the management of *T. absoluta* without negatively impacting the mirid predator associated with the pest.

## Data availability statement

The original contributions presented in the study are included in the article/**Supplementary Material**. Further inquiries can be directed to the corresponding authors.

## Author contributions

FK, BT, and BA conceptualized and designed the study. BA conducted the experiments, analyzed the data, and wrote the first draft of the manuscript. FK, BT, AY, and BA revised the manuscript and approved the final draft. FK and BT sourced funding for the study. All authors contributed to the article and approved the submitted version.

## References

[B1] Adams R. P. (1995). Terpenoid/Natural product library, a. r identification of essential oil components by gas chromatography/mass spectrometry (Carol Stream: Allured).

[B2] Ali T. O. Husin B. (2017). Biological control of tomato leaf miner tuta absoluta using entomopathogenic nematodes (United Kingdom: Newcastle University). Available at: http://theses.ncl.ac.uk/jspui/handle/10443/3845.

[B3] Antonio L. Gomes A. Carlos A. (2010). Resistance to the south American tomato pinworm *Tuta absoluta* in high acylsugar and / or high zingiberene tomato genotypes. Euphytica, 176, 113–123. doi: 10.1007/s10681-010-0234-8

[B4] Antwi-Agyakwa A. K. Fombong A. T. Deletre E. Ekesi S. Yusuf A. A. Pirk C.. (2019). Lemon terpenes influence behavior of the African citrus triozid *Trioza erytreae* (Hemiptera: Triozidae). J. Chem. Ecol. 45, 934–945. doi: 10.1007/s10886-019-01123-y 31755021

[B5] Antwi-Agyakwa A. K. Yusuf A. A. Pirk C. W. W. Mohamed S. A. Ekesi S. Torto B. (2021). Exploring non-host plant-based management strategy with lemongrass, garlic and guava volatiles for the African citrus triozid. J. Appl. Entomol. 1–10. doi: 10.1111/jen.12884

[B6] Ayelo P. M. Yusuf A. A. Pirk C. W. W. Chailleux A. Mohamed S. A. Deletre E. (2021). Terpenes from herbivore-induced tomato plant volatiles attract *Nesidiocoris tenuis* (Hemiptera: Miridae), a predator of major tomato pests. Pest Manage. Sci. 77, 5255–5267. doi: 10.1002/ps.6568 34310838

[B7] Bai P. H. Wang H. M. Liu B. S. Li M. Liu B. M. Gu X. S.. (2020). Botanical volatiles selection in mediating electrophysiological responses and reproductive behaviors for the fall webworm moth *Hyphantria cunea* . Front. Physiol. 11. doi: 10.3389/fphys.2020.00486 PMC7273966 32547409

[B8] Bankevich A. Nurk S. Antipov D. Gurevich A. A. Dvorkin M. Kulikov A. S.. (2012). SPAdes: A new genome assembly algorithm and its applications to single-cell sequencing. J. Comput. Biol. 19 (5), 455–477. doi: 10.1089/cmb.2012.0021 22506599 PMC3342519

[B9] Bawin T. Dujeu D. De Backer L. Francis F. Verheggen F. J. (2016). Ability of *Tuta absoluta* (Lepidoptera: Gelechiidae) to develop on alternative host plant species. Can. Entomol. 148, 434–442. doi: 10.4039/tce.2015.59

[B10] Benelli G. Govindarajan M. Rajeswary M. Vaseeharan B. Alyahya S. A. Alharbi N. S.. (2018). Insecticidal activity of camphene, zerumbone and α-humulene from *Cheilocostus speciosus* rhizome essential oil against the old-world bollworm, *Helicoverpa armigera* . Ecotoxicol. Environ. Saf. 148, 781–786. doi: 10.1016/j.ecoenv.2017.11.044 29190597

[B11] Biondi A. Guedes R. N. C. Wan F. H. Desneux N. (2018). Ecology, worldwide spread, and management of the invasive south American tomato pinworm, *Tuta absoluta*: Past, present, and future. Annu. Rev. Entomol. 63, 239–258. doi: 10.1146/annurev-ento-031616-034933 28977774

[B12] Bitew M. K. (2018). Significant role of wild genotypes of tomato trichomes for *Tuta absoluta* resistance. J. Plant Genet. Breed. 2, 1–12.

[B13] Camacho C. Coulouris G. Avagyan V. Ma N. Papadopoulos J. Bealer K.. (2009). BLAST+: Architecture and applications. BMC Bioinf. 10, 1–9. doi: 10.1186/1471-2105-10-421 PMC2803857 20003500

[B14] Campbell B. C. Steffen-Campbell J. D. Werren J. H. (1994). Phylogeny of the *Nasonia* species complex (Hymenoptera: Pteromalidae) inferred from an internal transcribed spacer (ITS2) and 28S rDNA sequences. Insect Mol. Biol. 2, 225–237. doi: 10.1111/j.1365-2583.1994.tb00142.x 9087560

[B15] Castañé C. Alomar O. Goula M. Gabarra R. (2004). Colonization of tomato greenhouses by the predatory mirid bugs *Macrolophus caliginosus* and *Dicyphus tamaninii* . Biol. Control 30, 591–597. doi: 10.1016/j.biocontrol.2004.02.012

[B16] Christofoli M. Costa E. C. C. Bicalho K. U. de Cássia Domingues V. Peixoto M. F. Alves C. C. F. (2015). Insecticidal effect of nanoencapsulated essential oils from *Zanthoxylum rhoifolium* (Rutaceae) in *Bemisia tabaci* populations. Ind. Crops Prod. 70, 301–308. doi: 10.1016/j.indcrop.2015.03.025

[B17] Cocco A. Deliperi S. Delrio G. (2013). Control of *Tuta absoluta* (Meyrick) (Lepidoptera: Gelechiidae) in greenhouse tomato crops using the mating disruption technique. J. Appl. Entomol. 137, 16–28. doi: 10.1111/j.1439-0418.2012.01735.x

[B18] Conboy N. J. A. McDaniel T. Ormerod A. George D. Gatehouse A. M. R. Wharton E. (2019). Companion planting with French marigolds protects tomato plants from glasshouse whiteflies through the emission of airborne limonene. PloS One 14, 1–21. doi: 10.1371/journal.pone.0213071 PMC6396911 30822326

[B19] Dardouri T. Gomez L. Ameline A. Costagliola G. Schoeny A. Gautier H. (2021). Non-host volatiles disturb the feeding behavior and reduce the fecundity of the green peach aphid, *Myzus persicae* . Pest Manage. Sci. 77, 1705–1713. doi: 10.1002/ps.6190 33200872

[B20] Desneux N. Wajnberg E. Wyckhuys K. A. G. Burgio G. Arpaia S. Narváez-Vasquez C. A.. (2010). Biological invasion of European tomato crops by *Tuta absoluta*: Ecology, geographic expansion and prospects for biological control. J. Pest Sci. 83, 197–215. doi: 10.1007/s10340-010-0321-6

[B21] Diabate S. Martin T. Murungi L. K. Fiaboe K. K. M. Subramanian S. Wesonga J.. (2019). Repellent activity of cymbopogon citratus and tagetes minuta and their specific volatiles against megalurothrips sjostedti. J. Appl. Entomol. 143, 855–866. doi: 10.1111/jen.12651

[B22] Erasmus R. Van Den Berg J. (2021). Susceptibility of *Tuta absoluta* (Lepidoptera: Gelechiidae) pupae to soil applied entomopathogenic fungal biopesticides. Insects 2021, 1–12. doi: 10.3390/insects12060515 PMC8228705 34199395

[B23] Fabrick J. A. Yool A. J. Spurgeon D. W. (2020). Insecticidal activity of marigold *Tagetes patula* plants and foliar extracts against the hemipteran pests, *Lygus hesperus* and *Bemisia tabaci* . PloS One 15, 1–15. doi: 10.1371/journal.pone.0233511 PMC7237031 32428032

[B24] Folmer O. Black M. Hoeh W. Lutz R. Vrijenhoek R. (1994). DNA Primers for amplification of mitochondrial cytochrome c oxidase subunit I from diverse metazoan invertebrates 0. Mol. Mar. Biol. Biotechnol. 14, 294–297. doi: 10.1071/ZO9660275 7881515

[B25] Giorgini M. Guerrieri E. Cascone P. Gontijo L. (2019). Current strategies and future outlook for managing the neotropical tomato pest *Tuta absoluta* (Meyrick) in the Mediterranean basin. Neotrop. Entomol. 48, 1–17. doi: 10.1007/s13744-018-0636-1 30284151

[B26] Goolsby J. A. De Barro P. J. Makinson J. R. Pemberton R. W. Hartley D. M. Frohlich D. R. (2006). Matching the origin of an invasive weed for selection of a herbivore haplotype for a biological control programme. Mol. Ecol. 15, 287–297. doi: 10.1111/j.1365-294X.2005.02788.x 16367847

[B27] Grez A. A. Zaviezo T. Gardiner M. M. (2014). Local predator composition and landscape affects biological control of aphids in alfalfa fields. Biol. Control 76, 1–9. doi: 10.1016/j.biocontrol.2014.04.005

[B28] Guedes R. N. C. Picanço M. C. (2012). The tomato borer *Tuta absoluta* in south America: pest status, management and insecticide resistance. EPPO Bull. 42, 211–216. doi: 10.1111/EPP.2557

[B29] Hassaballa I. B. Torto B. Sole C. L. Tchouassi D. P. (2021). Exploring the influence of different habitats and their volatile chemistry in modulating sand fly population structure in a leishmaniasis endemic foci, Kenya. PloS Negl. Trop. Dis. 15, e0009062. doi: 10.1371/JOURNAL.PNTD.0009062 33524028 PMC7877749

[B30] Iamba K. Homband V. (2020). Intercropping of pak choi (*Brassica rapa* chinensis) with marigold flower (*Tagetes erecta* l.) and onion (*Allium cepa* l.) to control foliar pests. J. Entomol. Zool. Stud. 8, 731–737.

[B31] Illakwahhi D. T. Srivastava P. B. (2017). Control and management of tomato leafminer -*Tuta absoluta* (Meyrick) (Lepidotera, gelechiidae). a review. IOSR J. Appl. Chem. 10, 14–22. doi: 10.9790/5736-1006011422

[B32] Kihika R. Tchouassi D. P. Ng’Ang’A M. M. Hall D. R. Beck J. J. Torto B. (2020). Compounds associated with infection by the root-knot nematode, *Meloidogyne javanica*, influence the ability of infective juveniles to recognize host plants. J. Agric. Food Chem. 68, 9100–9109. doi: 10.1021/acs.jafc.0c03386 32786872

[B33] Kihika-Opanda R. Tchouassi D. P. Ng’ang’a M. M. Beck J. J. Torto B. (2022). Chemo-ecological insights into the use of the non-host plant vegetable black-jack to protect two susceptible solanaceous crops from root-knot nematode parasitism. J. Agric. Food Chem. doi: 10.1021/ACS.JAFC.2C01748 35613461

[B34] Konan K. A. J. Monticelli L. S. Ouali-N’goran S. W. M. Ramirez-Romero R. Martin T. Desneux N. (2021). Combination of generalist predators, *Nesidiocoris tenuis* and *Macrolophus pygmaeus*, with a companion plant, *Sesamum indicum*: What benefit for biological control of *Tuta absoluta* ? PloS One 16, e0257925. doi: 10.1371/JOURNAL.PONE.0257925 34591899 PMC8483325

[B35] Lê Cao K. A. Boitard S. Besse P. (2011). Sparse PLS discriminant analysis: Biologically relevant feature selection and graphical displays for multiclass problems. BMC Bioinf. 12, 1471–2105. doi: 10.1186/1471-2105-12-253 PMC3133555 21693065

[B36] Liaw A. Wiener M. (2002). Classification and regression by randomForest. R News 2, 18–22.

[B37] Lins J. C. van Loon J. J. A. Bueno V. H. P. Lucas-Barbosa D. Dicke M. van Lenteren J. C. (2014). Response of the zoophytophagous predators *Macrolophus pygmaeus* and n*esidiocoris tenuis* to volatiles of uninfested plants and to plants infested by prey or conspecifics. BioControl 59, 707–718. doi: 10.1007/S10526-014-9602-Y/FIGURES/5

[B38] Liu H. Guo S. S. Lu L. Li D. Liang J. Huang Z. H.. (2021). Essential oil from *Artemisia annua* aerial parts: composition and repellent activity against two storage pests. Nat. Prod. Res. 35, 822–825. doi: 10.1080/14786419.2019.1599887 30961365

[B39] Matu F. K. Murungi L. K. Mohamed S. Deletre E. (2021). Behavioral response of the greenhouse whitefly (*Trialeurodes vaporariorum*) to plant volatiles of *Ocimum basilicum* and *Tagetes minuta* . Chemoecology 31, 47–62. doi: 10.1007/s00049-020-00327-z

[B40] Medeiros M. A. Sujii E. R. Morais H. C. (2009). Effect of plant diversification on abundance of south American tomato pinworm and predators in two cropping systems. Hortic. Bras. 27, 300–306. doi: 10.1590/S0102-05362009000300007

[B41] Miano R. N. Ayelo P. M. Mohamed S. A. (2022). Electroantennogram and machine learning reveal a volatile blend mediating avoidance behavior by *Tuta absoluta* females to a wild tomato plant. Sci. Rep. 12, 8965. doi: 10.1038/s41598-022-13125-0 35624177 PMC9142488

[B42] Moerkens R. Berckmoes E. Van Damme V. Ortega-Parra N. Hanssen I. Wuytack M.. (2016). High population densities of *Macrolophus pygmaeus* on tomato plants can cause economic fruit damage: Interaction with pepino mosaic virus? Pest Manage. Sci. 72, 1350–1358. doi: 10.1002/ps.4159 26419416

[B43] Msisi D. Matojo N. D. Kimbokota F. (2020). Attraction of female tomato leaf miner, *Tuta absoluta* (Meyrick 1917) (Lepidoptera:Gelechiidae) to shared compounds from hosts. Phytoparasitica. doi: 10.1007/s12600-020-00848-x

[B44] Mwamba S. Kihika-Opanda R. Murungi L. K. Losenge T. Beck J. J. Torto B. (2021). Identification of repellents from four non-host asteraceae plants for the root knot nematode, *Meloidogyne incognita* . J. Agric. Food Chem. 69, 15145–15156. doi: 10.1021/acs.jafc.1c06500 34882384

[B45] Naselli M. Zappalà L. Gugliuzzo A. Tropea Garzia G. Biondi A. Rapisarda C.. (2017). Olfactory response of the zoophytophagous mirid *Nesidiocoris tenuis* to tomato and alternative host plants. Arthropod. Plant Interact. 11 (2), 121–131. doi: 10.1007/s11829-016-9481-5

[B46] National Institute of Standards and Technology (2008) NIST/EPA/ NIH mass spectral library. natl. inst. stand. technol. NIST. Available at: http://www.nist.gov.

[B47] Njuguna P. K. Murungi L. K. Fombong A. Teal P. E. A. Beck J. J. Torto B. (2018). Cucumber and tomato volatiles: influence on attraction in the melon fly *Zeugodacus cucurbitate* (Diptera: Tephritidae). J. Agric. Food Chem. 66, 8504–8513. doi: 10.1021/acs.jafc.8b03452 30041516

[B48] Perdikis D. Favas C. Lykouressis D. Fantinou A. (2007). Ecological relationships between non-cultivated plants and insect predators in agroecosystems: the case of *Dittrichia viscosa* (Asteraceae) and *Macrolophus melanotoma* (Hemiptera: Miridae). Acta Oecologica 31, 299–306. doi: 10.1016/j.actao.2006.12.005

[B49] Piersanti S. Rebora M. Ederli L. Pasqualini S. Salerno G. (2020). Role of chemical cues in cabbage stink bug host plant selection. J. Insect Physiol. 120, 103994. doi: 10.1016/j.jinsphys.2019.103994 31830466

[B50] Rakha M. Zekeya N. Sevgan S. Musembi M. Ramasamy S. Hanson P. (2017). Screening recently identified whitefly/spider mite-resistant wild tomato accessions for resistance to *Tuta absoluta* . Plant Breed. 136, 562–568. doi: 10.1111/pbr.12503

[B51] Ranganathan Y. Borges R. M. (2010). Reducing the babel in plant volatile communication: Using the forest to see the trees. Plant Biol. 12, 735–742. doi: 10.1111/j.1438-8677.2009.00278.x 20701696

[B52] R Core Team (2018) The r project for statistical computing. Available at: https://www.r-project.org/ (Accessed June 7, 2022).

[B53] Rohart F. Gautier B. Singh A. Lê Cao K. A. (2017). mixOmics: An r package for ‘omics feature selection and multiple data integration. PloS Comput. Biol. 13, 1–19. doi: 10.1371/journal.pcbi.1005752 PMC5687754 29099853

[B54] Sanchez J. A. Cenis J. L. Cassis G. Martinez-Cascales J. I. (2006). Species identity of *Macrolophus melanotoma* (Costa 1853) and *Macrolophus pygmaeus* (Rambur 1839) (Insecta: Heteroptera: Miridae) based on morphological and molecular data and bionomic implications. Insect Systematics Evol. 37 (4), 385–404. doi: 10.1163/187631206788831470

[B55] Sanchez J. A. Lacasa A. Arnó J. Castañ C. Alomar O. (2009). Life history parameters for *Nesidiocoris tenuis* (Reuter) (Het., miridae) under different temperature regimes. J. Appl. Entomol. 133, 125–132. doi: 10.1111/j.1439-0418.2008.01342.x

[B56] Sanchez J. A. La-Spina M. Lacasa A. (2014). Numerical response of nesidiocoris tenuis (Hemiptera: Miridae) preying on *Tuta absoluta* (Lepidoptera: Gelechiidae) in tomato crops. Eur. J. Entomol. 111, 387–395. doi: 10.14411/eje.2014.041

[B57] Sanchez J. A. López-Gallego E. Pérez-Marcos M. Perera-Fernández L. G. Ramírez-Soria M. J. (2018). How safe is it to rely on *Macrolophus pygmaeus* (Hemiptera: Miridae) as a biocontrol agent in tomato crops? Front. Ecol. Evol. 6. doi: 10.3389/FEVO.2018.00132/BIBTEX

[B58] Silva D. B. Weldegergis B. T. Van Loon J. J. A. Bueno V. H. P. (2017). Qualitative and quantitative differences in herbivore-induced plant volatile blends from tomato plants infested by either *Tuta absoluta* or *Bemisia tabaci* . J. Chem. Ecol. 43, 53–65. doi: 10.1007/s10886-016-0807-7 28050733 PMC5331093

[B59] Sridhar V. Onkara naik S. Nitin K. Ashokan R. Swathi P. Gadad H. (2019). Efficacy of integrated pest management tools evaluated against *Tuta absoluta* (Meyrick) on tomato in India. J. Biol. Control 33, 264–270. doi: 10.18311/jbc/2019/23254

[B60] Subramani V. Pagadala Damodaram K. J. Goravale Krishnegowda R. Parepally S. K. Kempraj V. Thimmappa R.. (2021). Volatile chemical signals underlying the host plant preferences of *Tuta absoluta* . Entomol. Exp. Appl., 1–11. doi: 10.1111/eea.13099

[B61] Sylla S. Brévault T. Diarra K. Bearez P. Desneux N. (2016). Life-history traits of *Macrolophus pygmaeus* with different prey foods. PloS One 11, e0166610. doi: 10.1371/JOURNAL.PONE.0166610 27870857 PMC5117678

[B62] Tang G. B. Song B. Z. Zhao L. L. Sang X. S. Wan H. H. Zhang J.. (2013). Repellent and attractive effects of herbs on insects in pear orchards intercropped with aromatic plants. Agrofor. Syst. 87, 273–285. doi: 10.1007/s10457-012-9544-2

[B63] Tarusikirwa V. L. Machekano H. Mutamiswa R. Chidawanyika F. Nyamukondiwa C. (2020). *Tuta absoluta* (Meyrick) (lepidoptera: Gelechiidae) on the “offensive” in Africa: Prospects for integrated management initiatives. Insects 11, 1–33. doi: 10.3390/insects11110764 PMC7694550 33171892

[B64] Tembo Y. Mkindi A. G. Mkenda P. A. Mpumi N. Mwanauta R. Stevenson P. C. (2018). Pesticidal plant extracts improve yield and reduce insect pests on legume crops without harming beneficial arthropods. Front. Plant Sci. 9. doi: 10.3389/FPLS.2018.01425/BIBTEX PMC6172852 30323823

[B65] Tomova B. S. Waterhouse J. S. Doberski J. (2005). The effect of fractionated tagetes oil volatiles on aphid reproduction. Entomol. Exp. Appl. 115, 153–159. doi: 10.1111/J.1570-7458.2005.00291.X

[B66] Tonnang H. E. Z. Mohamed S. F. Khamis F. Ekesi S. (2015). Identification and risk assessment for worldwide invasion and spread of *Tuta absoluta* with a focus on Sub-Saharan Africa: Implications for phytosanitary measures and management. PloS One 10, 1–19. doi: 10.1371/journal.pone.0135283 PMC4529269 26252204

[B67] Torto B. Haukeland S. Cortada L. Coyne D. Murungi L. (2018). Management of cyst and root knot nematodes: A chemical ecology perspective. J. Agric. Food Chem. 66, 8672–8678. doi: 10.1021/acs.jafc.8b01940 30037217

[B68] Umpiérrez M. L. Lagreca M. E. Cabrera R. Grille G. Rossini C. (2012). Essential oils from asteraceae as potential biocontrol tools for tomato pests and diseases. Phytochem. Rev. 11, 339–350. doi: 10.1007/s11101-012-9253-5

[B69] van den Dool H. Dec. Kratz P. (1963). A generalization of the retention index system including linear temperature programmed gas–liquid partition chromatography. J. Chromatogr. A 11, 463–471. doi: 10.1016/S0021-9673(01)80947-X 14062605

[B70] van Lenteren J. C. (2012). The state of commercial augmentative biological control: Plenty of natural enemies, but a frustrating lack of uptake. BioControl 57, 1–20. doi: 10.1007/S10526-011-9395-1

[B71] Wang F. Park Y. L. Gutensohn M. (2021). Glandular trichome-derived mono- and sesquiterpenes of tomato have contrasting roles in the interaction with the potato aphid *Macrosiphum euphorbiae* . J. Chem. Ecol. 47, 204–214. doi: 10.1007/s10886-021-01243-4 33447946

[B72] Zappalà L. Biondi A. Alma A. Al-Jboory I. J. Arnò J. Bayram A.. (2013). Natural enemies of the south American moth, *Tuta absoluta*, in Europe, north Africa and middle East, and their potential use in pest control strategies. J. Pest Sci. 86, 635–647. doi: 10.1007/s10340-013-0531-9

