# Supplementary material for: Non-host plant odors influence the tritrophic interaction between tomato, its foliar herbivore Tuta absoluta and mirid predator Nesidiocoris tenuis
Source: Front Plant Sci. 2023 Mar 23;14:1014865. doi: 10.3389/fpls.2023.1014865 (PMC10076674; doi:10.3389/fpls.2023.1014865)
Supplement: Supplementary file 1 [file Table_1.docx]

**SUPPLEMENTARY**

**Table 1.** Pairwise comparison of host and non-host plants of *Tuta absoluta*

| Experiment | Treatment pairing |
| --- | --- |
| A | control vs. control |
| B | control vs. cultivated tomato |
| C | control vs. nightshade |
| D | control vs. marigold |
| E | control vs. blackjack |
| F | control vs. wild tomato |
| G | control vs. cultivated tomato + marigold |
| H | control vs. cultivated tomato + blackjack |
| I | control vs. cultivated tomato + wild tomato |
| J | control vs. nightshade + marigold |
| K | control vs. nightshade + blackjack |
| L | control vs. nightshade + wild tomato |
| M | cultivated tomato vs. cultivated tomato + marigold |
| N | cultivated tomato vs. cultivated tomato +. blackjack |
| O | cultivated tomato vs. cultivated tomato + wild tomato |
| P | nightshade vs. nightshade + marigold |
| U | nightshade vs. nightshade + blackjack |
| V | nightshade vs. nightshade + wild tomato |

**Table 2.** Pairwise comparison of host and non-host plant synthetic standard blends

| Experiment | Treatment pairing |
| --- | --- |
| A | control vs. 7-HP (i.e. 2.8 ng camphene, 156.4 ng δ-2-carene, 120.1 ng α-phellandrene, 45.7 ng α-terpinene, 147.1 ng *p*-cymene, 15.8 ng terpinolene, 13.1 ng (*E*, *E*)-α-farnesene) |
| B | control vs. 7-HPx2 (i.e. 5.6 ng camphene, 312.8 ng δ-2-carene, 240.2 ng α-phellandrene, 91.4 ng α-terpinene, 294.2 ng *p*-cymene, 31.6 ng terpinolene, 26.2 ng (*E*, *E*)-α-farnesene) |
| C | control vs. 7-HP/2 (i.e. 1.4 ng camphene, 78.2 ng δ-2-carene, 60.5 ng α-phellandrene, 22.9 ng α-terpinene, 73.6 ng *p*-cymene, 7.9 ng terpinolene, 6.6 ng (*E*, *E*)-α-farnesene) |
| D | control vs. 6-HPM (i.e. 2.8 ng camphene, 156.4 ng δ-2-carene, 120.1 ng α-phellandrene, 45.7 ng α-terpinene, 147.1 ng *p*-cymene, 15.8 ng terpinolene) |
| E | control vs. 6-HPMx2 (i.e. 5.6 ng camphene, 312.8 ng δ-2-carene, 240.2 ng α-phellandrene, 91.4 ng α-terpinene, 294.2 ng *p*-cymene, 31.6 ng terpinolene) |
| F | control vs. 6-HPM/2 (i.e. 1.4 ng camphene, 78.2 ng δ-2-carene, 60.5 ng α-phellandrene, 22.9 ng α-terpinene, 73.6 ng *p*-cymene, 7.9 ng terpinolene) |
| G | control vs. 13.1 ng (*E*, *E*)-α-farnesene |
| H | control vs. 26.2 ng (*E*, *E*)-α-farnesene |
| I | control vs. 6.6 ng (*E*, *E*)-α-farnesene |
| J | 6-HPM vs. 13.1 ng (*E*, *E*)-α-farnesene |
| K | 6-HPM vs. 26.2 ng (*E*, *E*)-α-farnesene |
| L | 6-HPM vs. 6.6 ng (*E*, *E*)-α-farnesene |
| M | control vs. 7-NHP (i.e. 98.7 ng α-pinene, 26.7 ng limonene, 407.3 ng β-phellandrene, 2.3 ng (*E*)-β-ocimene, 12.5 ng β-elemene, 4.6 ng α-cedrene, 17.6 ng α-humulene) |
| N | control vs. 7-NHPx2 (i.e. 197.4 ng α-pinene, 53.4 ng limonene, 814.6 ng β-phellandrene, 4.6 ng (*E*)-β-ocimene, 25 ng β-elemene, 9.2 ng α-cedrene, 35.2 ng α-humulene) |
| O | control vs. 7-NHP/2 (i.e. 49.4 ng α-pinene, 13.4 ng limonene, 203.7 ng β-phellandrene, 1.2 ng (*E*)-β-ocimene, 6.3 ng β-elemene, 2.3 ng α-cedrene, 8.8 ng α-humulene) |
| P | control vs. 4-NHPM (i.e. 98.7 ng α-pinene, 26.7 ng limonene, 407.3 ng β-phellandrene, 2.3 ng (*E*)-β-ocimene) |
| Q | control vs. 4-NHPMx2 (i.e. 197.4 ng α-pinene, 53.4 ng limonene, 814.6 ng β-phellandrene, 4.6 ng (*E*)-β-ocimene) |
| R | control vs. 4-NHPM/2 (i.e. 49.4 ng α-pinene, 13.2 ng limonene, 202.7 ng β-phellandrene, 1.2 ng (*E*)-β-ocimene) |
| S | control vs. 3-NHPS (i.e. 12.5 ng β-elemene, 4.6 ng α-cedrene, 17.6 ng α-humulene) |
| T | control vs. 3-NHPSx2 (i.e. 25 ng β-elemene, 9.2 ng α-cedrene, 35.2 ng α-humulene) |
| U | control vs. 3-NHPS/2 (i.e. 6.3 ng β-elemene, 2.3 ng α-cedrene, 8.8 ng α-humulene) |
| V | 7-NHPx2 vs. 3-NHPS |
| W | 7-NHPx2 vs. 3-NHPSx2 |
| X | 7-NHPx2 vs. 3-NHPS/2 |
